# From prediction to performance: *In silico*-supported formulation of amorphous solid dispersions for PROTAC NX-2127

**DOI:** 10.1016/j.ijpx.2026.100663

**Published:** 2026-09-10

**Authors:** Alicia Stakemeier, Lukas Mild, Johannes Sievers, Jitka Kalasová, Kristina E. Steffens, Martin Kuentz, Christian Steinebach, Karl G. Wagner

**Affiliations:** a Department of Pharmaceutical Technology and Biopharmaceutics, University of Bonn, Gerhard-Domagk-Str. 3, 53121 Bonn, Germany; b Department of Pharmaceutical/Medicinal Chemistry, Institute of Pharmacy, University of Greifswald, Friedrich-Ludwig-Jahn-Straße 17, 17489 Greifswald, Germany; c Institute for Pharma Technology and Biotechnology, University of Applied Sciences and Arts Northwestern Switzerland, Hofackerstr. 30, 4132 Muttenz, Switzerland; d Department of Pharmaceutical Sciences, University of Basel, Klingenbergstrasse 50, 4052 Basel, Switzerland

**Keywords:** PROTAC, NX-2127, Amorphous solid dispersions, Supersaturation, Solubility enhancement, Ternary mixtures, Ionic crosslinking

## Abstract

The oral delivery of PROTACs is frequently limited by poor aqueous solubility and low membrane permeability, whereas early formulation development is often constrained by limited drug substance availability. To address these challenges, this study established a material-sparing workflow integrating *in silico* prediction with *in vitro* screening to efficiently support formulation development of the model PROTAC NX-2127. Following physicochemical characterization confirming the amorphous nature and thermal stability of NX-2127, COSMOquick calculations served as an initial tool to prioritize polymer candidates, while miniaturized supersaturation assay enabled their final selection for amorphous solid dispersions (ASDs). Soluplus® and Eudragit® EPO exhibited superior supersaturation-inducing and -maintaining properties and were selected for ASD preparation *via* vacuum compression molding (VCM). To further enhance dissolution, ionic crosslinking within the EPO-based ASD was induced by incorporating tartaric acid (TA), yielding a ternary EPO/TA-ASD. This formulation delivered a pronounced enhancement in non-sink dissolution, resulting in a 546-fold increase in drug release in phosphate buffer (pH 6.8) compared to the neat compound at the endpoint of the dissolution assay at 270 min. These findings were consistently reflected in the biorelevant biphasic dissolution system (BiPHa+), where the EPO/TAASD exhibited the highest performance and further improved partitioning into the absorption compartment. Overall, this study demonstrates that while *in silico* prediction represents a valuable tool for supporting early formulation development, experimental *in vitro* screening remains indispensable for reliable polymer selection. The combination of both approaches enabled the rational design of ASD formulations and led to substantial gains in dissolution performance of poorly soluble PROTACs.

## Graphical abstract

**Figure:**
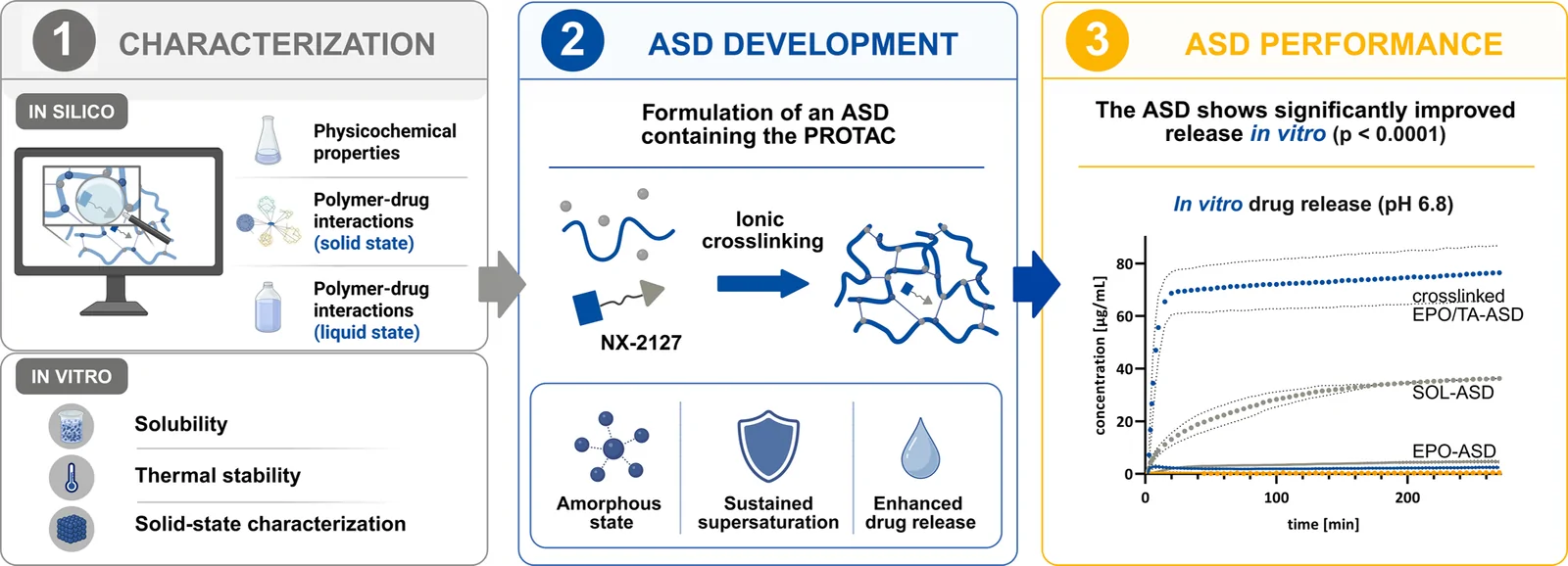

## 1 Introduction

PROteolysis Targeting Chimeras (PROTACs) represent a novel class of therapeutics that induce selective degradation of disease-relevant proteins of interest (POI) by hijacking the proteasome. These bifunctional molecules link the POI to an E3 ligase, commonly Von Hippel–Lindau (VHL) or cereblon, leading to ubiquitination and subsequent proteasomal degradation. Since their first description in 2001, over 50 PROTACs have entered clinical trials, with the first approval achieved in 2026 for Vepdegestrant (Neklesa et al., 2017; Fan et al., 2025; Sakamoto et al., 2001). Most PROTAC research focuses on oncology, including targets such as Bruton's tyrosine kinase (BTK), a key driver in B-cell malignancies like chronic lymphatic leukemia (CLL) and mantle cell lymphoma (MCL). Conventional BTK inhibitors, such as ibrutinib, often lose efficacy owing to resistance mutations. In contrast, the PROTAC NX-2127 (Zelebrudomide) promotes degradation of BTK and additionally targets the transcription factors IKZF-1 and IKZF-3, resulting in immunomodulatory effects (Wang et al., 2022; Kim, 2025; Robbins et al., 2024). During a Phase I study in relapsed CLL patients, NX-2127 achieved over 80% BTK degradation, accompanied by a 79% clinical response rate independent of mutational status, with generally manageable side effects. These findings highlight the therapeutic potential of PROTAC-based strategies (Montoya et al., 2024; Danilov et al., 2023).

The majority of PROTACs share certain characteristics due to their distinctive chemical composition. These characteristics include poor aqueous solubility, low permeability, off-target toxicity, and hook-effects (Syahputra et al., 2025; Edmondson et al., 2019; Yokoo et al., 2023). The molecular weight of prototypical cereblon-PROTACs is usually approximately 800 Da, with a range of 20–30 rotatable bonds, a calculated lipophilicity of 1–7, a polar surface area above 140 Å^2^ and a number of hydrogen bond donors of 3–4 (Yokoo et al., 2023; Poongavanam et al., 2023; Hornberger and Araujo, 2023). The high molecular weight of these substances has been referred to as ‘molecular obesity’, a term that captures the complexity of their composition (Syahputra et al., 2025; Hann, 2011). The ligand-linker-ligand structure frequently results in high polar surface area, leading to limited membrane permeability. In accordance with Lipinski's rule of five (Ro5) and Veber's rule, the majority of PROTACs are located outside the boundaries of the previously defined chemical spaces (Edmondson et al., 2019; Lipinski et al., 2012; Veber et al., 2002). As with NX-2127, the aqueous solubility and permeability postulated for this beyond rule of 5 (bRo5) substance are inadequate. A recent study reported an aqueous solubility of only 0.2 μM at pH 7.4 for NX-2127, along with a Caco-2 permeability of less than 0.2 * 10^−6^ cm/s and a high efflux ratio of >18 (Robbins et al., 2024; Srivastava et al., 2025). Notwithstanding, an oral bioavailability (BA) of 36% (Robbins et al., 2024) was determined in the mouse model. However, Robbins et al. (Robbins et al., 2024) could neither transfer this oral BA to dogs nor to cynomolgus monkeys. Subsequently, the oral bioavailability drops to only 1.0% and 1.2% in both non-rodent species (Robbins et al., 2024). Such a phenomenon is described in a comparative analysis of cynomolgus monkeys and rodent species, which revealed a lower apparent intestinal permeability (P_app_) in monkeys, which is often attributed to higher intestinal efflux transporter expression, stronger enterocytic metabolism, and lower apical uptake rates. Nevertheless, cynomolgus monkeys are more analogous to humans than rodents. Consequently, the use of rodent models may result in an overestimation of passive intestinal permeation (Takahashi et al., 2008; Musther et al., 2014; Kambayashi et al., 2022). Beyond the species-dependent differences observed *in vivo*, the complex absorption mechanisms and chameleonic properties of PROTACs also limit the predictive value of commonly used *in vitro* permeability assays, such as PAMPA and Caco-2, which often fail to reflect *in vivo* absorption behavior (Yokoo et al., 2023; Niessen et al., 2025; Niessen et al., 2026; Pike et al., 2020). Despite the rarity of existing literature on the formulation of the specific PROTAC NX-2127, a single study has reported on NX-2127 preparations using the principle of ASDs. Polymers such as HPMCAS, Povidone, and PEG have been suggested. However, no specific polymer was specified in the aforementioned study (Wang and Chen, n.d.). Given the limitations of available predictive permeability assays for PROTACs, the present study focuses exclusively on addressing the solubility issues of NX-2127 as a first step to potentially increase bioavailability, as dissolution is regarded as a necessary step prior to absorption (Robbins et al., 2024; Danilov et al., 2023). A variety of methods for enhancing solubility and permeability, while limiting off-target toxicity in the context of PROTACs, have previously been outlined in the research literature (Syahputra et al., 2025). Rathod et al. proposed a self-nanoemulsifying drug delivery system (SNEDDS) for the drug substance ARV-825, which stabilized the supersaturated state while maintaining a droplet size of 45.02 nm. However, a major limitation of this approach is the high amount of dimethylacetamide required, which is classified as a class 2 solvent by the ICH (Rathod et al., 2019; EMA, 2024). Both Saraswat et al. and Zhang et al. achieved improved permeation in cell-based assays using liposomal formulations prepared by the thin film hydration method (Saraswat et al., 2022; Zhang et al., 2023). As a further formulation approach, various PROTACs were embedded in polymeric or lipid nanoparticle matrices (Saraswat et al., 2020; He et al., 2022; Arenas-Moreira et al., 2025; Jeong et al., 2025). A critical review of these formulation principles revealed one common thread: they were only optimized for parenteral administration. Nevertheless, in order to enhance patient tolerance and simplify the dosing regimen, an oral form of therapy is frequently pursued. Amorphous solid dispersions (ASDs) are particularly well-suited for this purpose, as they have been extensively discussed for PROTAC formulations in the literature (Pöstges et al., 2023; Screen et al., 2025a; Hofmann et al., 2025; Hofmann et al., 2024). A molecular dispersion in an amorphous polymer stabilizes primarily kinetically the metastable amorphous state of the drug molecule in the solid state. In that way, solubility is dependent on the dissolution rate of the ASD particles and the ability of the ASD-forming polymer to stabilize the supersaturated state in solution (parachute effect). In case high supersaturation levels and maintenance of supersaturation cannot be provided by a single polymer, ternary ASDs comprising two polymers or other enabling excipients, *e.g.* surfactants, are employed (Sun and Lee, 2013; Lin et al., 2018; Chiou and Riegelman, 1971). Typically, the manufacturing process for these formulations involves the application of either spray drying (SD) or hot melt extrusion (HME). The drug substance is embedded either after nebulization from a polymer-containing solution through a nozzle or after dissolving in a molten polymeric carrier under shear action (Tambe et al., 2022; Vasconcelos et al., 2016; Leuner, 2000).

Considering the restricted accessibility of the substance NX-2127, screening methodologies based on SD or HME are deemed unfeasible in our experimental setup. To minimize drug substance amounts and maximize mechanistic information, we chose an approach in which, in a first step, ASD formation and stability in the solid state and maintenance of supersaturation were assessed *via* simulation (COSMOquick) (Price et al., 2019; Loschen and Klamt, 2012). In the second step, supersaturation maintenance was assessed experimentally in an assay similar to the determination of the amorphous solubility; however, using polymer-containing media (Pöstges et al., 2023; López Mármol et al., 2021). Consequently, microscale vacuum compression molding (VCM) was employed as a surrogate for HME, given that only a few milligrams of the active ingredient are required for this purpose (Shadambikar et al., 2020). The formulations were subjected to stability testing in order to evaluate their risk of phase separation during processing. Furthermore, a novel concept regarding the ionic crosslinking of alkaline polymers, such as Eudragit® EPO, as recently described by Mild et al. (Mild et al., 2026), *via* the addition of an organic acid within the ASD during HME was employed. This approach promotes ionic interactions within the polymer matrix, which are expected to reduce polymer chain mobility and thereby potentially enhance drug release and apparent solubility of NX-2127. The present study aimed to investigate the potential applicability of the newly proposed concept to poorly soluble PROTACs while simultaneously minimizing the consumption of drug substance during formulation development.

## 2 Materials and methods

### 2.1 Materials

**Fig. 1:**
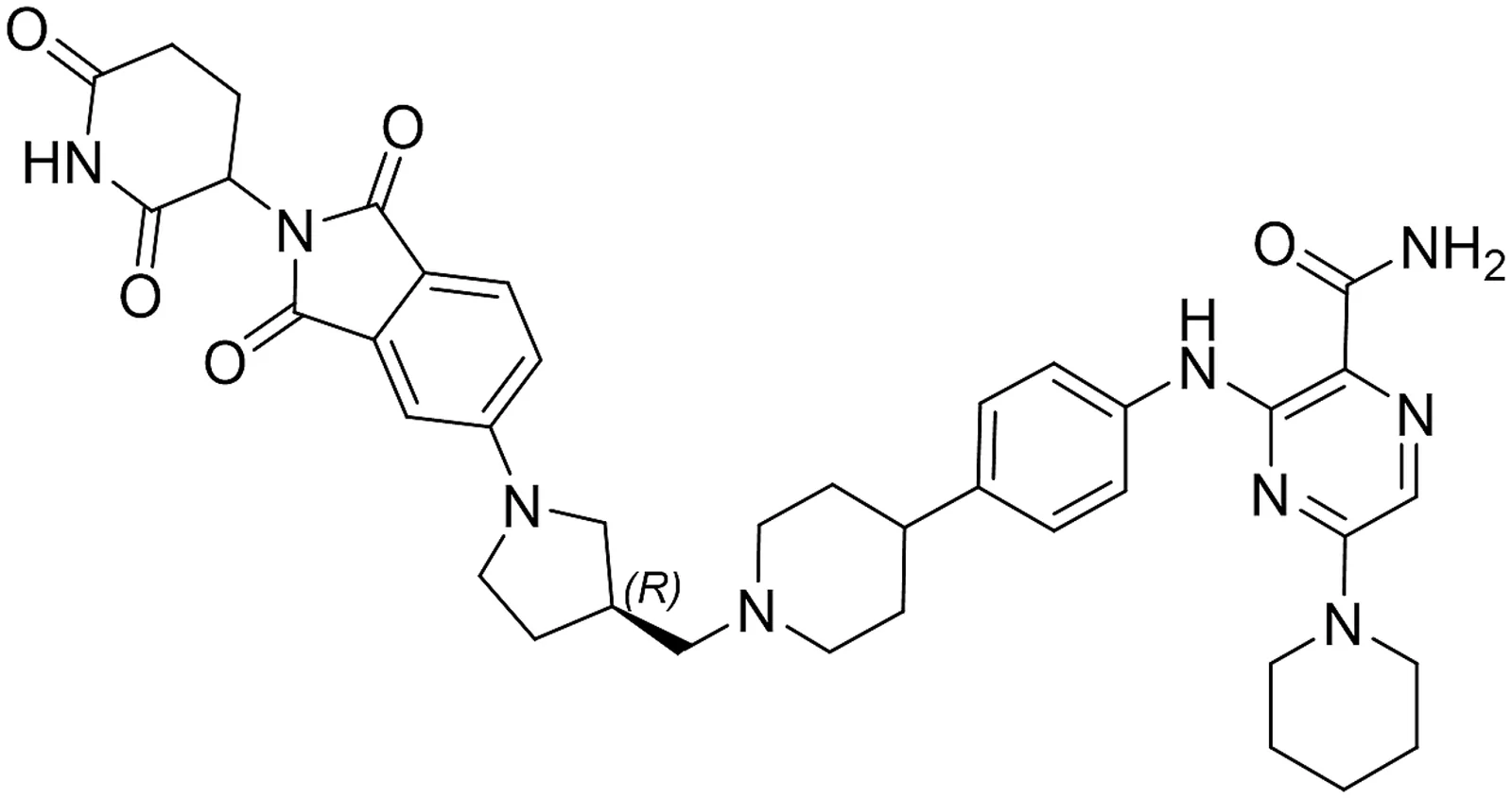
Chemical structure of studied PROTAC NX-2127 (M*w* = 720 g/mol).

The PROTAC NX-2127 (Fig. 1, Table 1) was synthesized in-house in our laboratories (section 2.1.1). AQOAT® (HPMCAS, L-Grade), HPMCP 55 and Metolose® SM (MC) were kindly gifted by ShinEtsu Chemical (Tokyo, Japan). HPC-SSL was sent from Nippon Soda Co., Ltd. (Tokyo, Japan), Eudragit® E PO (EPO), and Eudragit® L 100–55 from Evonik (Darmstadt, Germany), *L*-tartaric acid from Boehringer Ingelheim (Ingelheim, Germany), and HPMC HME 15 LV from DuPont Pharma & Nutrition (Luzern, Switzerland). Kolliphor® P188, povidone (Kollidon® 30), copovidone (Kollidon® VA64), and amphiphilic graft copolymer comprised of polyvinyl caprolactam, polyvinyl acetate and polyethylene glycol (Soluplus®, SOL) were kindly provided by BASF (Ludwigshafen, Germany). Polyethylene glycol 6000 and hydroxyethyl cellulose were obtained from Carl Roth GmbH (Karlsruhe, Germany). Dimethyl sulfoxide (DMSO, ≥ 99.9%) was obtained from TCI Deutschland GmbH (Eschborn, Germany), polyvinyl alcohol 115,000, decanol, acetonitrile, and methanol from VWR International S.A.S. (Rosny-sous-Bois, France). Disodium hydrogen phosphate dihydrate and sodium dihydrogen phosphate dihydrate were purchased from Th. Geyer (Renningen, Germany). The compositions of FaSSIF-V2 and FeSSIF-V1 are listed in the supplementary information (supplementary Data, Table S1). All chemicals used were of analytical grade.

**Table 1:** Physicochemical properties of PROTAC NX-2127 calculated using the ADMET Predictor 12.0.0.6 platform (Simulations Plus, Inc.).

Molecular weight *Mw*	720 g/mol
Predicted pKa (basic)	8.38
Predicted logP	3.4
Predicted water solubility	0.064 mg/mL
Predicted permeability (S + Peff)	0.407 cm/s *10^4^

#### 2.1.1 Synthesis of NX-2127

The convergent synthetic route for compound **9** (NX-2127) is outlined in Fig. S1. Synthesis of the eastern fragment commenced from 3,5-dichloropyrazine-2‑carbonitrile (Neklesa et al., 2017), whereas synthesis of the linker part started from (*S*)-1-(*tert*-butoxycarbonyl)pyrrolidine-3-carboxylic acid (Kim, 2025). Assembly of the target molecule involved a sequence of different transformations, including a nucleophilic aromatic substitution reaction, a Buchwald–Hartwig cross-coupling, synthesis and reduction of the Weinreb-amide **6**, reductive amination, and a final nucleophilic aromatic substitution step with commercially available 5-fluorothalidomide. Following final fragment coupling, compound **9** (NX-2127) was purified by flash column chromatography and subjected to repeated drying cycles to ensure efficient removal of residual solvents. Compound identity and purity were confirmed by NMR spectroscopy and LC-MS analysis as described in the supplementary data.

### 2.2 Preparation methods

Initially, 500 mg of several physical mixtures (PMs) of NX-2127 and the selected polymer in a ratio of 1:9 (10% drug load) were prepared by blending the raw materials using an oscillating mill (MM400, Retsch, Germany), which was loaded with glass beads and operated at a frequency of 30 Hz. Three milling cycles of five minutes were performed to obtain a homogeneous PM. The compositions of each formulation are listed in Table 2. Subsequently, the vacuum compression molding technique (VCM, MeltPrep GmbH, Graz, Austria) was used to prepare the amorphous solid dispersions (ASDs) (Treffer et al., 2015). Approximately 40 mg of the respective PM was introduced into the Teflon-coated chamber of the 8-mm VCM device. The samples were then subjected to vacuum heating for 15 min at 180 °C, which is above the glass transition temperature of the PROTAC. Rapid cooling of the melt using compressed air led to the formation of a solid ASD disc, which was then ground using an oscillating mill (MM400, Retsch, Germany) and passed through a 355 μm sieve to obtain a homogeneous particle size distribution.

**Table 2:** Composition of prepared formulations with an overall drug loading of 10% NX-2127 in various polymer matrices (batch size: 500 mg).

Formulation	Composition	Annealing temperature and time
ASD 1	NX-2127: Soluplus® (SOL) 10:90	180 °C, 15 min
ASD 2	NX-2127: Eudragit® EPO (EPO) 10:90	
ASD 3	NX-2127: Eudragit® EPO containing 20% tartaric acid 10:90	

### 2.3 Analytical and characterization methods

#### 2.3.1 X-ray powder diffraction (XRPD)

The solid state of neat NX-2127 and the milled ASDs was characterized using an X'Pert PRO MPD (PANalytical, Almelo, The Netherlands) at 45 kV and 40 mA equipped with an X-Celerator detector and CuKα1 radiation. Samples were enclosed using Mylar foil and PW1818/25-PW1818/40 sample holders. Transmission mode measurements were carried out within a 10–40° 2θ range. A step size of 0.017° 2θ and a scanning speed of 0.021°/s were applied to provide sufficient sensitivity for detecting trace crystallinity. All experiments were carried out in triplicate (*n* = 3).

#### 2.3.2 Thermal analysis

The decomposition profile of NX-2127 was determined using TGA/ SDTA 851e (Mettler, Gießen, Germany). The raw material was weighed in an aluminum oxide pan and heated from 25 °C to 600 °C (10 °C/ min) with a nitrogen purge of 20 mL/ min. The weight loss [%] was plotted against temperature in order to obtain the decomposition profile. Furthermore, DSC analysis was conducted using a DSC 2 calorimeter (Mettler, Gießen, Germany) equipped with a nitrogen cooling system and nitrogen as purge gas (30 mL/min). Measurements of the pure raw materials and the processed ASDs were carried out in TOPEM mode, a multi-frequency, temperature-modulated program with a heating rate of 2 °C/min, a temperature pulse amplitude of ±0.5 °C, and a variable pulse interval ranging from 15 to 30 s. Approximately 10 mg of each sample was accurately weighed into aluminum pans, which were sealed with a pierced lid. The temperature range was dependent on the previously determined decomposition profile of the sample and ranged from 25 °C to either 120 °C, 170 °C, or 200 °C. Each experiment was conducted in triplicate (*n* = 3).

#### 2.3.3 Polarized light microscopy (PLM)

Residual crystallinity of the prepared ASDs was determined using PLM images taken with a Leica DM 2700 M microscope (Leica microsystems, Wetzlar, Germany) equipped with a Q-Imaging MicroPublisher 5.0 RTV camera (Teledyne Photometrics, Tucson AZ, USA). Images were captured using Q-Capture Pro 7 software version 7.0.5. A small amount of the sample was placed directly onto a microscope slide. The polarizer and analyzer were positioned orthogonally to determine the birefringence, which indicates residual crystallinity. Each sample was analyzed in triplicate (*n* = 3).

#### 2.3.4 Fourier-transform infrared (FT-IR) spectroscopy

A Spectrum Two FT-IR spectrometer (PerkinElmer, Waltham, MA, USA) with an ATR (attenuated total reflectance) diamond module was used to examine solid-state molecular interactions. Accordingly, raw materials, physical mixtures, and ASDs were analyzed within a spectral range of 450–4000 cm^−1^ with a resolution of 1 cm^−1^. All experiments were done in triplicate (*n* = 3).

#### 2.3.5 Shaking flask assay

Saturation solubility was determined in 1 mL decanol, 0.1 N hydrochloric acid, 0.05 M phosphate buffer (pH 6.8 and 7.4), as well as biorelevant media (FaSSIF-V2 and FeSSIF-V1, composition is shown in Table S2) using the shake flask method. Decanol was included as a medium to evaluate whether sink conditions could be achieved in the absorption compartment of the subsequent BiPHa+ assay. An excess of NX-2127 was added to 5 mL of small glass vials and shaken at 37 °C for 48 h. Subsequently, the samples were centrifuged at 15.000 rpm for a duration of 15 min, followed by appropriate dilution to prevent precipitation. The quantification of dissolved NX-2127 was conducted using reversed-phase ultra-performance liquid chromatography coupled with an Acquity™ TQD mass spectrometer (LCMS, Waters GmbH, Eschborn, Germany). An RP18 column (Nucleodur, 125 mm × 4.6 mm, 3 μm, EC, Macherey Nagel, Düren, Germany) was used as a stationary phase. The mobile phase, composed of 0.5 mM ammonium acetate and acetonitrile with 0.1% formic acid, was utilized as a gradient from 70% to 30% 0.5 mM ammonium acetate within 15 min. A volume of 10 μL was injected at a flow rate of 0.3 mL/min. The ionization of NX-2127 was conducted through electron spray ionization in the positive ion mode, with detection at 720 *m*/*z* in the single ion recording mode. Calibration was performed within a range of 50 to 350 ng/mL.

#### 2.3.6 Second harmonic scattering (SHS)

The saturation solubility of NX-2127 in 0.05 M phosphate buffer (pH 6.8) was additionally determined using a recently published second harmonic scattering (SHS) method (Kalasová et al., 2025). NX-2127 was first dissolved in DMSO and then serially diluted to prepare 11 stock solutions at 100-fold the final test concentrations. Aliquots of 1 μL of each stock solution were added to 99 μL of phosphate buffer in individual wells of a 96 well-plate. A blank containing 1% (*v*/*v*) DMSO in the buffer was included for baseline determination. The plate was sealed with TiterTops® sealing film to minimize evaporation and incubated for 24 h prior to analysis. Samples were then measured using an ultrafast laser (1030 nm, 66.67 kHz, 350 fs; NKT Photonics A/S, Birkerød, Denmark). The SHS signal was collected and detected as described previously; full details of the optical setup and measurement principle are provided in a previous study (Kalasová et al., 2025). Solubility was assigned as the concentration at which the linearly interpolated SHS signal crossed the baseline +3σ threshold, with the baseline defined as the mean intensity of the blank and the two lowest tested concentrations. All experiments were performed in triplicate (*n* = 3).

#### 2.3.7 Particle size analysis

Particle size distributions were measured using a Horiba LA-960 laser diffractometer (Horiba Ltd., Kyoto, Japan). 15 mL of polymer solution (1.25 mg/mL) in 0.05 M phosphate buffer pH 6.8 was accurately filled into a quartz cuvette. Pre-dissolved NX-2127 in DMSO was spiked into the aqueous phase under continuous magnetic stirring to ensure a homogeneous distribution. The resulting particle size distributions were continuously measured for 180 min at predefined time points (0, 5, 10, 15, 30, 45, 60, 120, and 180 min). A red laser diode with a wavelength of 650 nm (5 mW) and a blue light-emitting diode with a wavelength of 405 nm (3 mW) were used as light sources. Volumetric particle size distributions were calculated using the Mie scattering model. All measurements were performed in triplicate (*n* = 3).

### 2.4 *In silico* methods

#### 2.4.1 COSMOquick ranking

The COSMOquick software (COSMOlogic, Germany, Version 2025) was utilized to simulate the interaction between NX-2127 and various polymers, as well as the drugs' self-cohesion. The calculation is based on the COSMO-RS theory, as described by Klamt et al. (Klamt, 1995). Drug-excipient calculations were performed under equimolar assumptions at 37 °C. Owing to the inherent complexity of long polymer chains, their structures were approximated as simplified oligomers in the so-called polymer mode. The potential for drug-excipient interactions was assessed using a rank-order parameter known as the enthalpy of interaction (Hex), otherwise referred to as the enthalpy change of mixing (ΔH_mix_) (Price et al., 2019; Loschen and Klamt, 2012).

(1) $$∆H_{\mathrm{mix}}=\sum_{k}x_{k}H_{\mathrm{mix}}^{k}-\sum_{k}x_{k}H_{\mathrm{pure}}^{k}$$

Equation 1: Calculation of ΔH_mix_.

#### 2.4.2 Principal component analysis (PCA) and partial least squares (PLS) regression

All cheminformatics calculations for the investigated polymers were performed using the open-source software RDKit (http://www.rdkit.org) in Python. The polymer structures were encoded in SMILES format and imported into RDKit to calculate more than 200 molecular descriptors (Landrum, 2013). To investigate relationships among these descriptors and identify the main sources of structural variation within the polymer dataset, PCA and PLS regression were conducted using SIMCA 18.2 (Sartorius Stedim Data Analytics AB, Umeå, Sweden). Prior to analysis, all descriptor variables were mean-centered and unit variance scaled. PCA was used to explore the descriptor patterns and structural similarities among the polymers using loading and score plots. Descriptors exhibiting high absolute loading values were considered the primary contributors to the respective principal components. PLS regression was subsequently applied to identify the molecular descriptors associated with polymer interactions with NX-2127, resulting in the maximum supersaturated concentration. The contribution of individual descriptors was evaluated using loading values and variable importance plots (VIP).

### 2.5 *In vitro* methods

#### 2.5.1 Supersaturation assay for polymer screening

A supersaturation assay was performed to determine the appropriate polymer for stabilizing supersaturation. Only a few milligrams of sample are required to perform this assay. Various 10 mL solutions of polymers, each with a concentration of 1.25 mg/mL in 0.05 M phosphate buffer (pH 6.8), were prepared and stirred at a temperature of 37 °C with a rotational speed of 240 rpm in small glass vials. Subsequently, 50 μL of a 40 mg/mL NX-2127 stock solution in dimethyl sulfoxide (DMSO) was added. Samples were collected at 30 and 60 min and quantitatively analyzed using reversed-phase high-performance liquid chromatography (HPLC, Agilent Technologies Deutschland GmbH, Waldbronn, Germany) coupled with a diode array detector (DAD). The elution process was carried out isocratically within 20 min, using a mobile phase consisting of methanol and 0.1% formic acid in water, at a ratio of 80% to 20%, with a flow rate of 1 mL/min. The injection volume was set to 100 μL to reduce the limit of quantification. An RP18-Nucleosil-column (250 mm × 4.6 mm, 5 μm, EC, Macherey Nagel, Düren, Germany) was used as a stationary phase. Calibration was performed within a range of 5.5 to 44 μg/mL with UV detection at 308 nm.

#### 2.5.2 Supersaturation assay

To assess the polymer's capacity to stabilize the achieved supersaturation over an adequate timeframe, the supersaturation assay was conducted in an automated configuration. 50 mL of the respective 1.25 mg/mL polymer solution in 0.05 M phosphate buffer pH 6.8 was prepared at 37 °C and 240 rpm. To this, 250 μL of a 40 mg/mL DMSO stock solution was added. Since ASD formulations with drug loads of 10% and 20% were initially planned, the polymer concentration was selected as a compromise to represent the NX-2127: polymer ratio of both drug load levels, corresponding to a theoretical ASD drug load of 14% (Pöstges et al., 2023). Owing to limited material availability, only the 10% drug load ASD was investigated in the subsequent studies. The amount of NX-2127 that dissolved was continuously determined using an 8453 UV/ VIS DAD spectrophotometer (Agilent Technologies Deutschland GmbH, Waldbronn, Germany) at 308 nm for 270 min. Scattering due to precipitation during the assay was corrected by subtracting the mean measured absorbance between 500 and 550 nm. Additionally, the results were verified by a HPLC endpoint control. The method outlined in section 2.5.1 was employed for this purpose.

#### 2.5.3 Non-sink dissolution testing

The dissolution of selected ASD powders, listed in Table 2, was examined to evaluate their capacity to enhance apparent solubility in an intestine-mimicking medium. To minimize sample sizes, the miniaturized USP dissolution apparatus II (MiniDissolution apparatus) was utilized (Zecevic and Wagner, 2013). The dissolution of several PM and ASD was conducted in 20 mL of 0.05 M phosphate buffer (pH 6.8) at 37 °C with a paddle rotation speed of 75 rpm. A precise amount of 40 mg of the formulation was accurately weighed into each vessel, resulting in a maximum concentration of 0.2 mg/mL per vessel. Released NX-2127 was quantified in-line using an 8453 UV/ VIS DAD spectrophotometer (Agilent Technologies Deutschland GmbH, Waldbronn, Germany) at a wavelength of 308 nm over a period of 270 min. The measured data underwent scattering correction, as previously described, and were validated *via* an HPLC endpoint control (see section 2.5.1). This process is intended to eliminate particle scattering resulting from precipitation or the potential self-absorption of certain polymers, such as SOL. Dissolution testing was performed in triplicate (*n* = 3).

#### 2.5.4 Biorelevant biphasic dissolution (BiPHa+)

A selection of ASD formulations was examined using a biphasic dissolution setup developed by Denninger et al. (Denninger et al., 2020). The assay was designed to assess the performance of the formulation as it passed through the human gastrointestinal tract, including passive absorption. The BiPHa+-Setup consists of four cylindrical vessels placed in a water bath maintained at 37 °C with a rotational speed of 160 rpm to ensure optimal hydrodynamics. During the initial 30-min period, 50 mL of 0.1 N hydrochloric acid (pH 1.0) was introduced into each vessel to simulate the conditions of the stomach. This was followed by the addition of a FaSSIF and a buffer concentrate, which had previously been developed by Denninger et al. (Denninger et al., 2020). The addition of bile salts, as well as shifting the pH to 5.5, mimicked transit through the upper small intestine. Furthermore, the aqueous phase was overlaid with a 50 mL decanol layer to create a drug absorption compartment. Over the following 60 min, the pH value was incrementally elevated to 6.8, thereby simulating the subsequent passage through the small intestine. To ascertain the quantity of NX-2127 released, samples were collected from both the aqueous and organic phases at predetermined time points (30, 60, 120, 180, and 270 min). Following adequate dilution, the samples were quantified using the HPLC method outlined in section 2.5.1.

### 2.6 Statistical analysis

Data are presented as mean ± standard deviation (SD) from three independent experiments (*n* = 3). Statistical analysis was performed using one-way analysis of variance (ANOVA) followed by Dunnett's multiple comparisons test to compare the ability to maintain supersaturation of each formulation introduced in Table 2 with the aqueous solubility of NX-2127 at pH 6.8 (control). Differences were considered statistically significant at *p* < 0.05 (* < 0.05, ** < 0.01, *** < 0.001, **** < 0.0001). All analyses were performed using GraphPad Prism version 8.4.0.

## 3 Results

### 3.1 Physicochemical characterization of pure PROTAC NX-2127

#### 3.1.1 Solid State of NX-2127

**Fig. 2:**
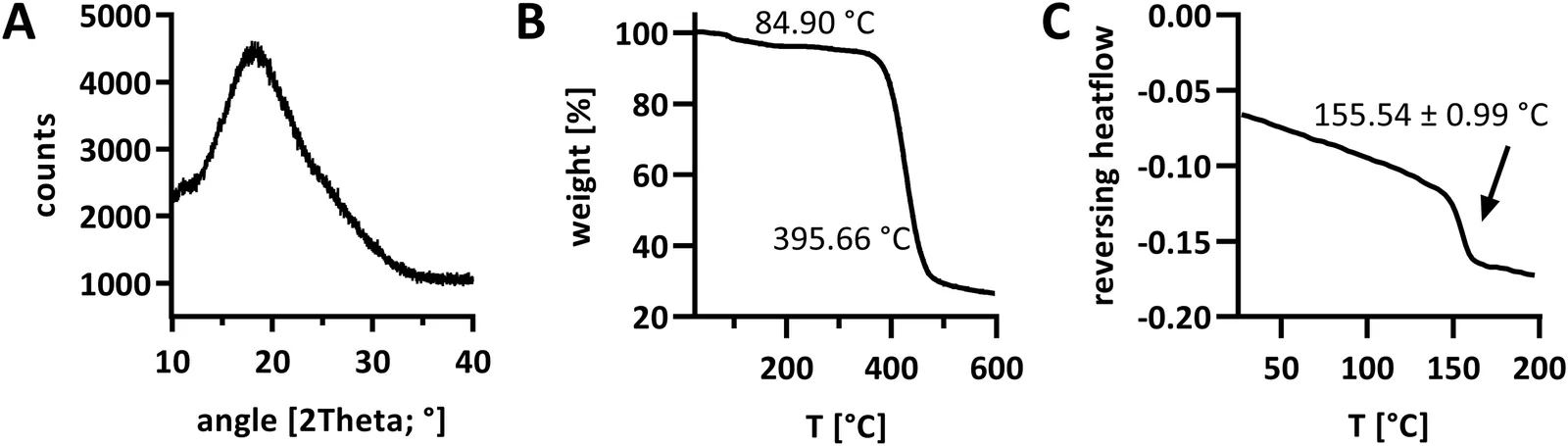
Solid state analysis of pure NX-2127: (A) X-ray powder diffraction (XRPD) pattern of pure NX-2127; (B) Thermal stability of NX-2127 determined by thermogravimetric analysis (TGA); (C) Differential scanning calorimetry (DSC) thermogram of pure NX-2127 (exo up).

The XRPD diffractogram of pure NX-2127 showed no detectable Bragg reflections, characteristic of amorphous materials (Fig. 2A). The resulting diffraction pattern showed a characteristic amorphous halo, indicating the absence of crystalline structure. This observation was further supported by DSC analysis, which showed no melting peak but only a glass transition, consistent with an amorphous solid state (Fig. 2C). Specifically, the glass transition of 155.54 ± 0.99 °C was determined for NX-2127.

Thermal stability was additionally assessed by TGA, which indicated stability up to 395.66 °C (Fig. 2B). A minor mass loss of 1.26% was observed at 84.9 °C, which was attributed to residual water due to the amorphous character of the drug substance or residual solvents from the synthesis (Fig. 2B). Furthermore, the chemical stability of NX-2127 was evaluated by LC-MS after heating at 200 °C for 15 min. We detected a minimal decomposition of the substance resulting in less than 2% loss in purity, which confirmed the thermal stability (data not shown).

#### 3.1.2 Saturation solubility

The saturation solubility of NX-2127 was determined using two complementary approaches: the conventional shake-flask method and the recently developed second harmonic scattering (SHS) technique. The shake-flask assay was performed at three different pH values, revealing a clear pH-dependent solubility profile. The highest thermodynamic solubility was observed in 0.1 N HCl, with a measured concentration of 0.398 ± 0.057 μg/mL. At a pH of 6.8, the solubility remained at 0.140 ± 0.049 μg/mL, whereas increasing the pH to 7.4 resulted in a marked decrease to 0.068 ± 0.006 μg/mL (Table 3).

**Table 3:** Saturation solubility of NX-2127 in different biorelevant media determined either using shake-flask assay in a small scale (1 mL) or using second harmonic scattering as described in section 2.3.6 (labeled with ‘#’). Quantification was performed using LC-MS analysis as outlined in section 2.3.5.

Medium	Saturation solubility [μg/mL]
0.1 N HCl pH 1	0.398 ± 0.057
Phosphate buffer pH 6.8	0.140 ± 0.049
Phosphate buffer pH 6.8 ^#^	0,122 ± 0.05
Phosphate buffer pH 7.4	0.068 ± 0.006
FaSSIF-V2 pH 6.5	0.159 ± 0.012
FeSSIF-V1 pH 5	7.791 ± 0.189
Decanol	573.756 ± 8.590

In addition, the solubility was assessed in biorelevant media. In FaSSIF-V2, the thermodynamic solubility of NX-2127 was 0.159 ± 0.012 μg/mL, whereas in FeSSIF-V1, a 49-fold increase was observed, reaching 7.791 ± 0.189 μg/mL. This marked increase in solubility in FeSSIF-V1 indicates a potential positive food effect, as the higher bile salt and phospholipid concentrations present under fed-state conditions enhance the solubilization of poorly water-soluble compounds, *e.g.* PROTACs (Pike et al., 2020). Moreover, the solubility of NX-2127 in decanol was determined to be 573.756 ± 8.590 μg/mL, suggesting that sink conditions in the absorption compartment of the BiPHa+ assay could be adequately maintained.

**Fig. 3:**
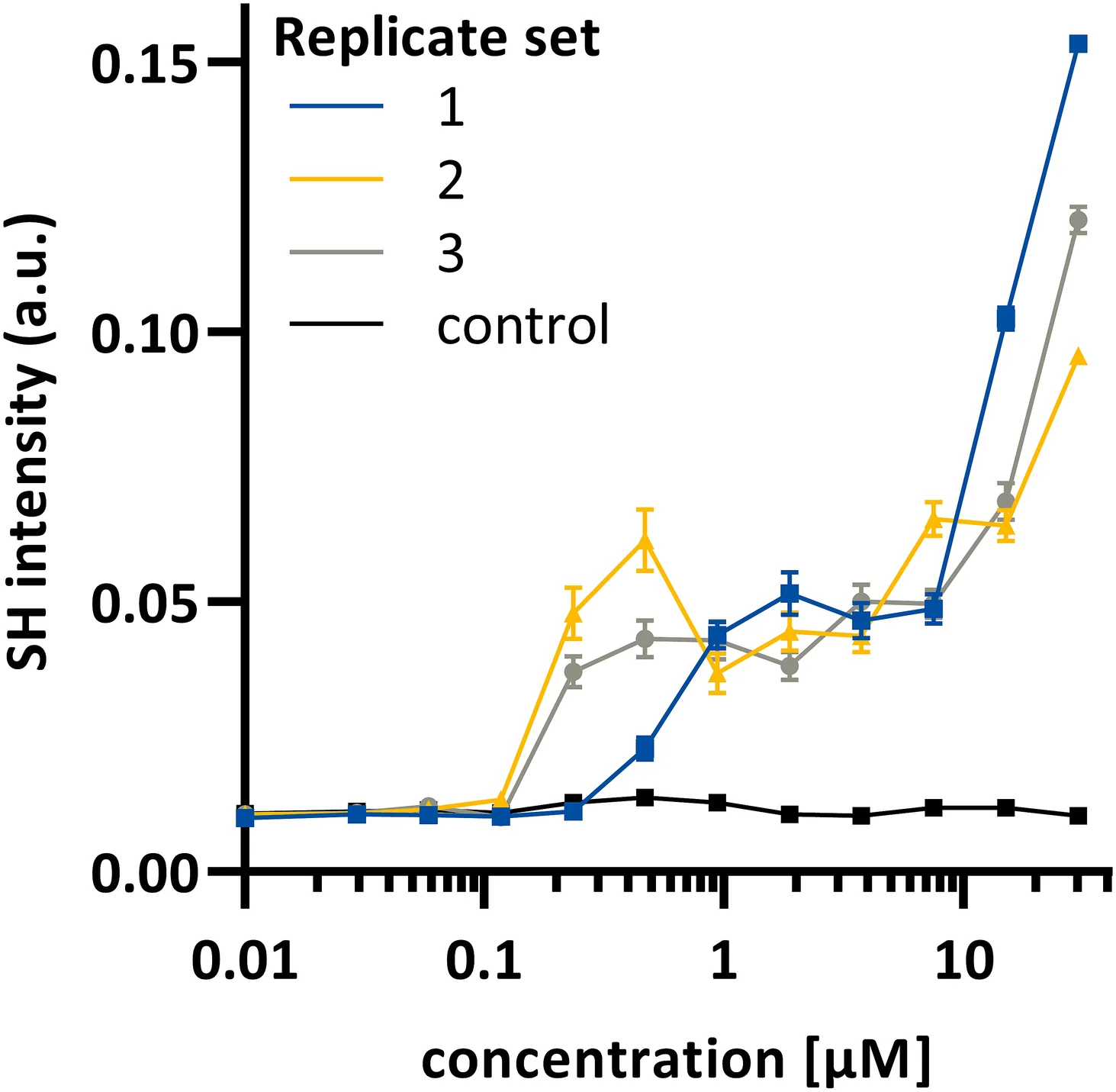
SH intensity as a function of NX-2127 concentrations in a range between 0.01 and 30 μM to determine the solubility of NX-2127 in 100 μL of 0.05 M phosphate buffer pH 6.8 after 24 h. The plot shows data from three independent replicate sets (shown in blue, yellow and green) and a negative control (highly water-soluble caffeine, shown in grey). Error bars represent the variability of across 30 measurements of a single sample.

The saturation solubility at pH 6.8 was also determined using SHS. Fig. 3 shows the SH intensity profile of NX-2127 at concentrations ranging from 0.01 to 30 μM. At low concentrations (0.01–0.1 μM), below the solubility limit, the SH intensity signal remained stable. Above this threshold, a pronounced increase in SH intensity was observed with increasing concentration, indicating the onset of molecular clustering and aggregate formation, which disrupts dipole symmetry and enhances SH response. This behavior is characteristic of non-centrosymmetric molecular assemblies. Based on this transition, the solubility limit of NX-2127 at pH 6.8 was determined to be 0.170 ± 0.07 μM (0.122 ± 0.05 μg/mL).

### 3.2 Polymer screening

#### 3.2.1 COSMOquick ranking

**Fig. 4:**
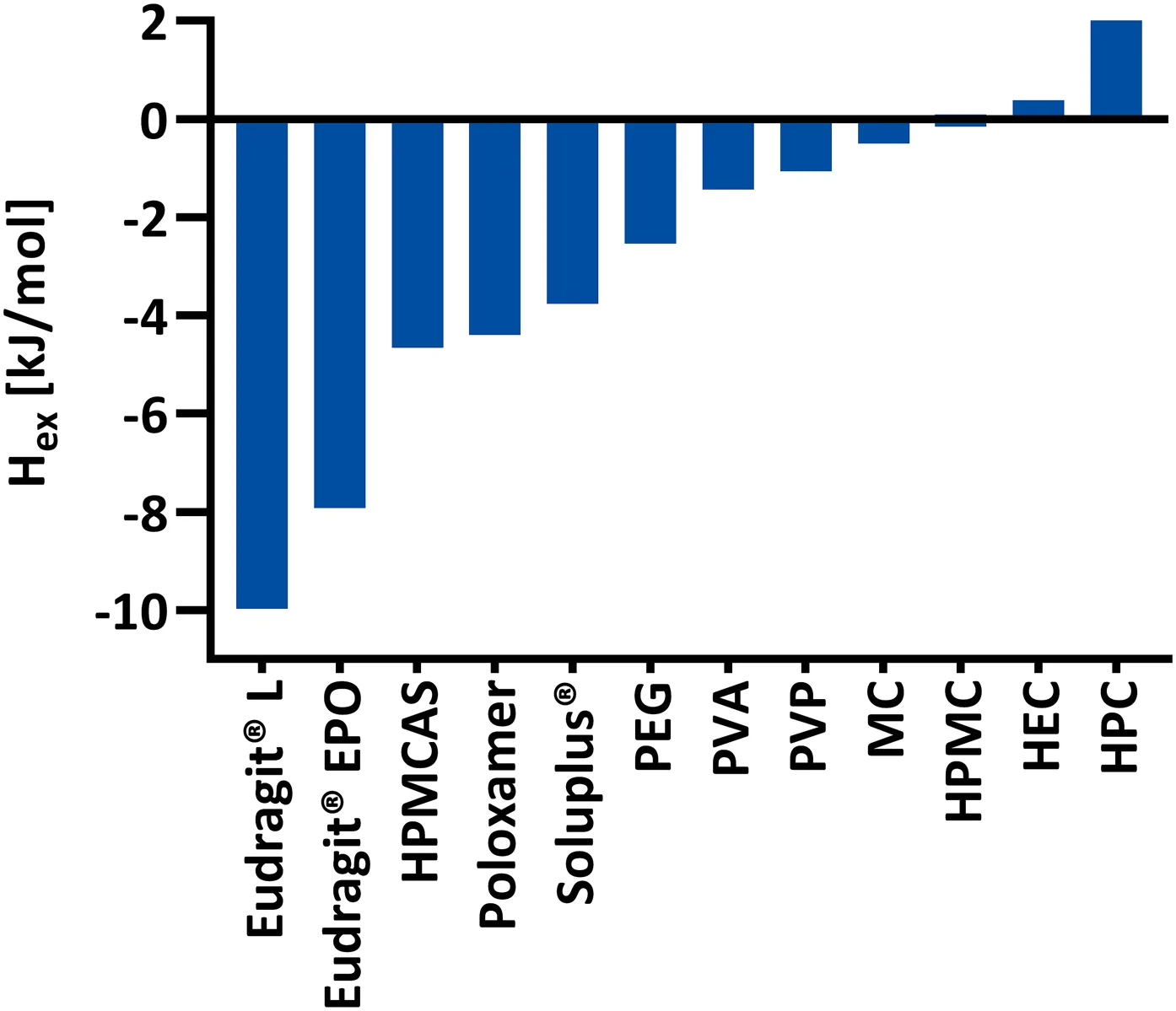
COSMOS-RS Rank: calculated excess enthalpy of interaction between PROTAC NX-2127 with several precipitation inhibiting polymers: Eudragit® L, Eudragit® EPO, hydroxypropylmethylcellulose acetate succinate (HPMCAS), Poloxamer, Soluplus®, polyethylenglycol (PEG), polyvinylalcohol (PVA), polyvinylpyrrolidone (PVP), methylcellulose (MC), hydroxypropylmethylcellulose (HPMC), hydroxyethylcellulose (HEC), hydroxypropylcellulose (HPC). Values are given in kJ/mol and ranked from high to low theoretical potential to stabilize supersaturation.

The COSMO-RS *in silico* screening was conducted by calculating the enthalpy of interaction between NX-2127 and a range of potential ASD polymers and precipitation inhibitors. Primarily, a low mixing enthalpy likely indicates a high solubility of NX-2127 in the respective polymer. However, an extrapolation of the pure drug/polymer-interaction into an aqueous liquid phase was suggested by various authors, with more negative interaction enthalpies corresponding to an increased ability of the polymer to inhibit API precipitation in aqueous media (Price et al., 2019; Loschen and Klamt, 2012; Klamt, 1995). Although COSMO-RS is capable of describing solvation effects *via* an implicit thermodynamic framework, it does not explicitly account for solvent molecules or dynamic solution behavior. Consequently, the applied screening approach primarily reflects binary molecular interactions (*e.g.*, API-polymer) and provides only a simplified representation of the actual solution environment (Theilacker et al., 2015). Among the investigated excipients, Eudragit® L and EPO demonstrated the most favorable performance as precipitation inhibitors (Fig. 4). Furthermore, HPMCAS, Poloxamer, and Soluplus® exhibited moderately negative mixing enthalpies (approximately −4 kJ/mol), indicating weak but potentially relevant interactions that warrant further *in vitro* investigation. In contrast, cellulose derivatives, including methylcellulose (MC), hydroxypropylmethylcellulose (HPMC), hydroxyethylcellulose (HEC) and hydroxypropylcellulose (HPC), were predicted to be less suitable as precipitation inhibitors. Notably, HPC showed a positive mixing enthalpy of +2.00 kJ/mol, suggesting unfavorable interactions and a lack of stabilizing potential.

#### 3.2.2 Supersaturation assay

**Fig. 5:**
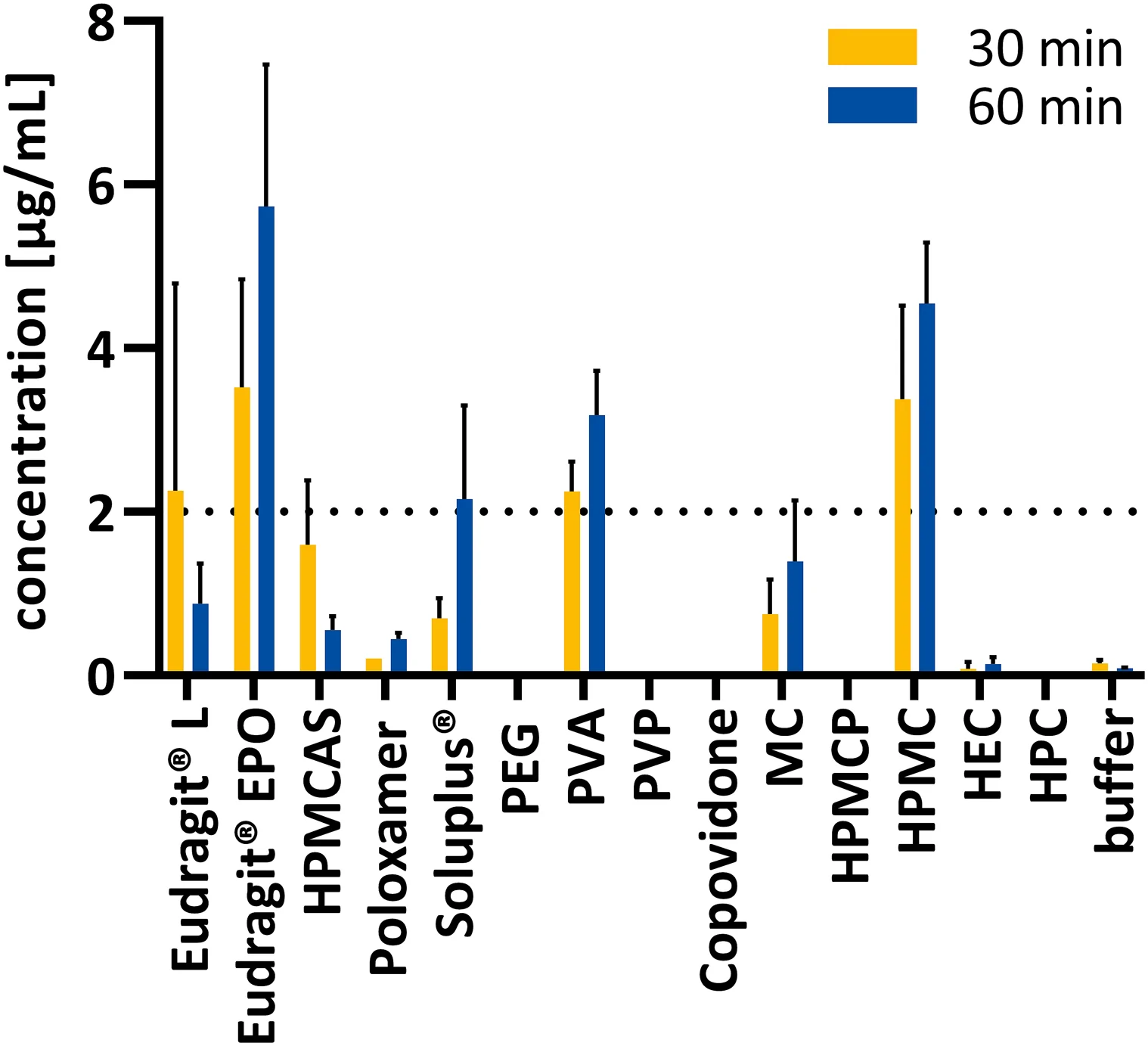
Supersaturation assay of 0.2 mg/mL NX-2127 in 10 mL 0.05 M phosphate buffer at pH 6.8 (37 °C, 240 rpm) with various pre-dissolved polymers (Eudragit® L, Eudragit® EPO, hydroxypropylmethylcellulose acetate succinate (HPMCAS), Poloxamer, Soluplus®, polyethylenglycol (PEG), polyvinylalcohol (PVA), polyvinylpyrrolidone (PVP), methylcellulose (MC), hydroxypropylmethylcellulose (HPMC), hydroxyethylcellulose (HEC), hydroxypropylcellulose (HPC))at a concentration of 1.25 mg/mL. The plot shows drug concentrations after 30 min in yellow bars and after 60 min in blue bars.

For corroboration of the COSMOquick ranking, the supersaturation assay was performed to identify the polymer most effective at stabilizing supersaturation. Subsequently, a more detailed evaluation was conducted for all polymers that achieved supersaturation levels above 2 μg/mL, as visualized by the dashed line in Fig. 5. Eudragit® L and HPMCAS were excluded from further analysis due to their inability to maintain supersaturation for 60 min, despite reaching supersaturation within the first 30 min.

**Fig. 6:**
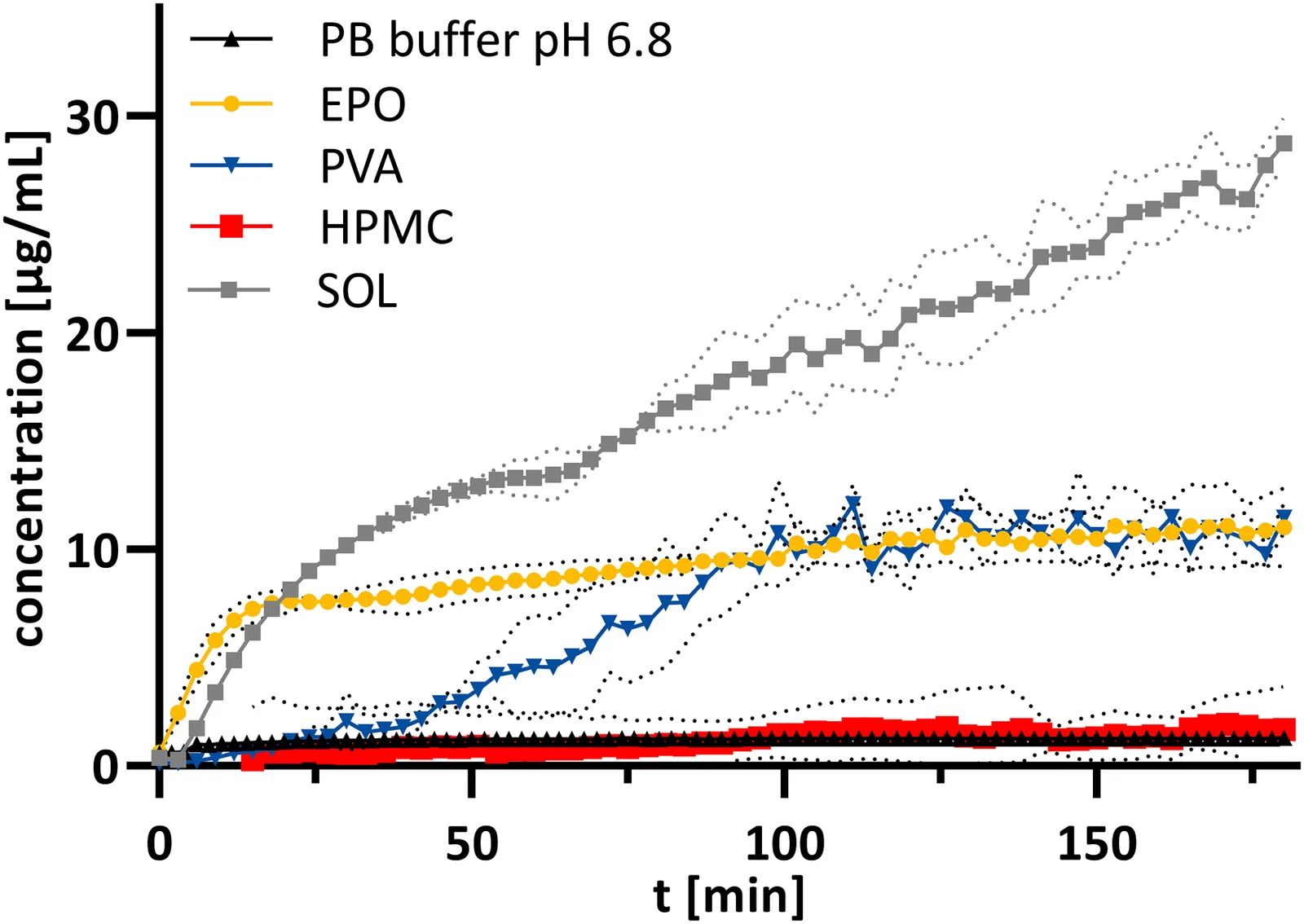
Kinetics during the supersaturation assay of 0.2 mg/mL NX-2127 in 50 mL 0.05 M phosphate buffer at pH 6.8 (37 °C, 240 rpm) with different pre-dissolved polymers at a concentration of 1.25 mg/mL: ▲ without polymer,  SOL,  EPO,  PVA and  HPMC.

Based on the polymer screening results (Fig. 5), a focused investigation was carried out for SOL, EPO, PVA and HPMC, as these systems were able to sustain supersaturation levels above 2 μg/mL. Over 180 min, SOL exhibited the highest supersaturation levels, followed by EPO and PVA, respectively (Fig. 6). In the case of SOL, the concentration-time profile suggested a two-stage distribution process for NX-2127 following the spiking of the DMSO stock solution into the polymer medium. During the initial 60 min, the concentration increased to a plateau of 14 μg/mL. Thereafter, a second redistribution phase occurred, with the concentration increasing to 29.12 ± 3.35 μg/mL by the end of the experiment, although a steady-state concentration could not be confirmed within the experimental timeframe. Since the literature describes SOL as micelle-forming, this behavior may be attributed to partial partitioning of the PROTAC into SOL micelles (Guembe-Michel et al., 2025). Even in the presence of pre-dissolved EPO, NX-2127 released from the spiked DMSO stock solution was not immediately available but instead reached a supersaturation plateau of 11.02 ± 0.61 μg/mL after 10 min. Concentrations were confirmed *via* HPLC. Particle formation during the experiments was additionally assessed by laser diffraction. As the metastable supersaturated state developed, a decrease in particle size was observed. In the SOL system, a bimodal particle size distribution with a fraction below 100 nm became apparent after 45 min (Supplementary Data, Fig. S2, Table S2). In contrast, PVA only achieved supersaturation of NX-2127 after a delay of 100 min and was therefore excluded from further consideration. HPMC did not reach supersaturation under the applied conditions and was likewise excluded. Consequently, SOL and EPO were selected for further investigation in the context of ASD formation. As the present study predominantly focused on the capacity of the polymers to maintain supersaturation, a more detailed assessment of their ability to form stable ASDs is warranted.

### 3.3 Characterization of prepared amorphous solid dispersions (ASDs)

#### 3.3.1 Solid state characterization of prepared ASDs

**Fig. 7:**
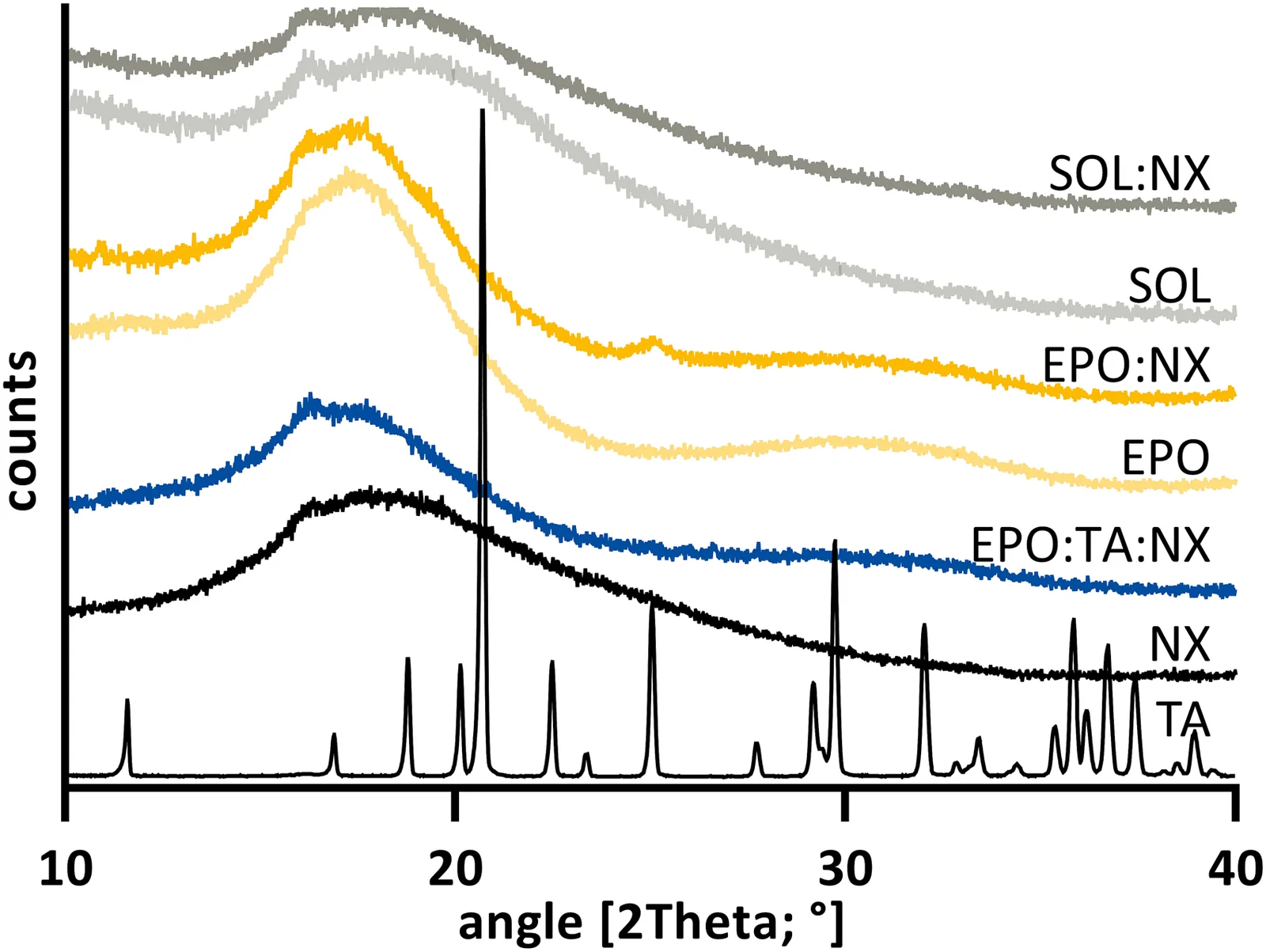
X-ray powder diffraction (XRPD) pattern of pure raw materials and 10% drug-loaded ASDs:  SOL (ASD 1) and NX-2127,  EPO (ASD 2) and NX-2127, as well as  EPO + TA (ASD 3) and NX-2127 measured in transmission mode with an angle from 10 to 40° 2θ. All samples were milled prior to analysis and measured in triplicate (*n* = 3). A representative spectrum is shown. Diffractograms of physical mixtures can be found in supplementary materials (Fig. S3).

The solid-state properties of the prepared ASDs were characterized by X-ray powder diffraction (XRPD), polarized light microscopy (PLM) and differential scanning calorimetry (DSC). The XRPD patterns of the pure PROTAC NX-2127 and the corresponding ASD formulations are shown in Fig. 7. As the amorphous character of pure NX-2127 had already been confirmed by XRPD (section 3.1.1), the absence of sharp Bragg reflections in the binary ASDs containing SOL or EPO was expected. In contrast, the absence of crystalline reflections in the ternary EPO/TA-ASD is particularly noteworthy, as TA is crystalline in its initial state. These results suggest that TA was converted into an amorphous state within the ASD. PLM further supported these findings, as no birefringent regions were observed in any of the formulations, indicating the absence of detectable residual crystallinity (Supplementary Data, Fig. S4).

**Fig. 8:**
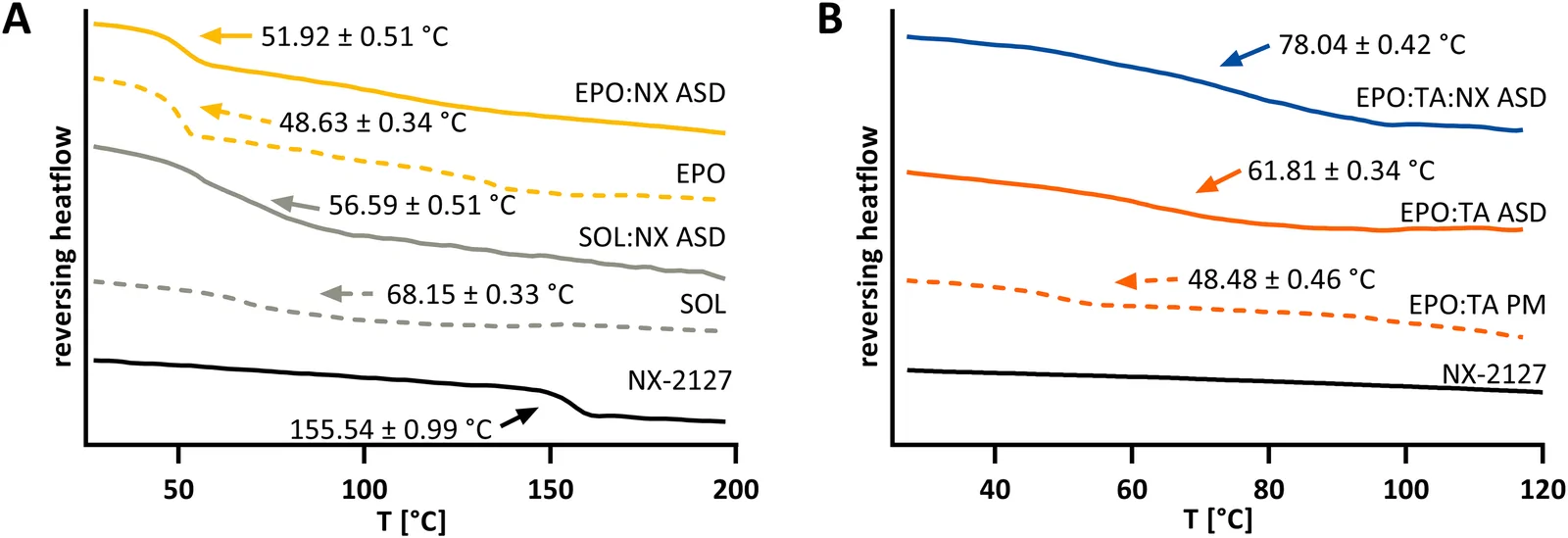
TOPEM differential scanning calorimetry (DSC) thermograms (exo up) of NX-2127, pure polymers and 10% drug-loaded ASDs of A:  EPO and  SOL heated from 25 to 200 °C and B:  10% drug-loaded ASD of EPO with 20% tartaric acid and  EPO with 20% tartaric acid without NX-2127 ASD heated from 25 to 120 °C. Values are represented as mean ± SD (*n* = 3).

Additionally, DSC analysis confirmed these findings, as no melting peak was detected in any of the formulations (Fig. 8). In the case of SOL, the glass transition temperature (T*g*) exhibited a notable decrease of 14 °C after the formation of an ASD, reaching 56.59 ± 0.51 °C compared to the neat SOL. In contrast, the binary ASD consisting of EPO and NX-2127 exhibited a marginal increase in T*g* from 48.63 ± 0.34 °C in the case of pure polymer to 51.92 ± 0.51 °C for the ASD. Conversely, the ternary ASD comprising EPO, NX-2127, and TA exhibited a distinct mixed T*g* of 78.04 ± 5.42 °C. A similar trend is observed in the binary ASD consisting of EPO and TA (mixed T*g* for the binary ASD at 61.81 ± 0.34 °C) compared to the physical mixture.

To further assess the miscibility of the ASDs, the experimentally determined T*g*'s were compared with the values predicted by the Gordon-Taylor (GT) (Gordon and Taylor, 1952) and Couchman-Karasz (CK) equation (Couchman and Karasz, 1978; Bochmann et al., 2016) (Supplementary Fig. S5). The binary SOL/NX-2127 and EPO/NX-2127 ASDs exhibited lower T*g* values than predicted (SOL-ASD: – 20.30 °C (GT); −29.14 °C (CK), EPO-ASD: - 7.40 °C (GT); −13.01 °C (CK)), suggesting enhanced molecular mobility compared to that expected from ideal mixing. This behavior may arise from plasticization by the amorphous drug or residual moisture, as well as weaker specific polymer-drug interactions that assumed by the Gordon-Taylor or Couchman-Karasz equation. In contrast, the ternary EPO/TA/NX-2127 ASD exhibited a T*g* that exceeded both predictions (+ 6.86 °C (GT); + 5.31 °C (CK)), indicating a formation of specific intermolecular interactions, most likely ionic interactions between EPO, TA and NX-2127. This interaction can potentially restrict molecular mobility, resulting in a higher T*g* value.

#### 3.3.2 Intermolecular interactions between NX-2127 and polymer

**Fig. 9:**
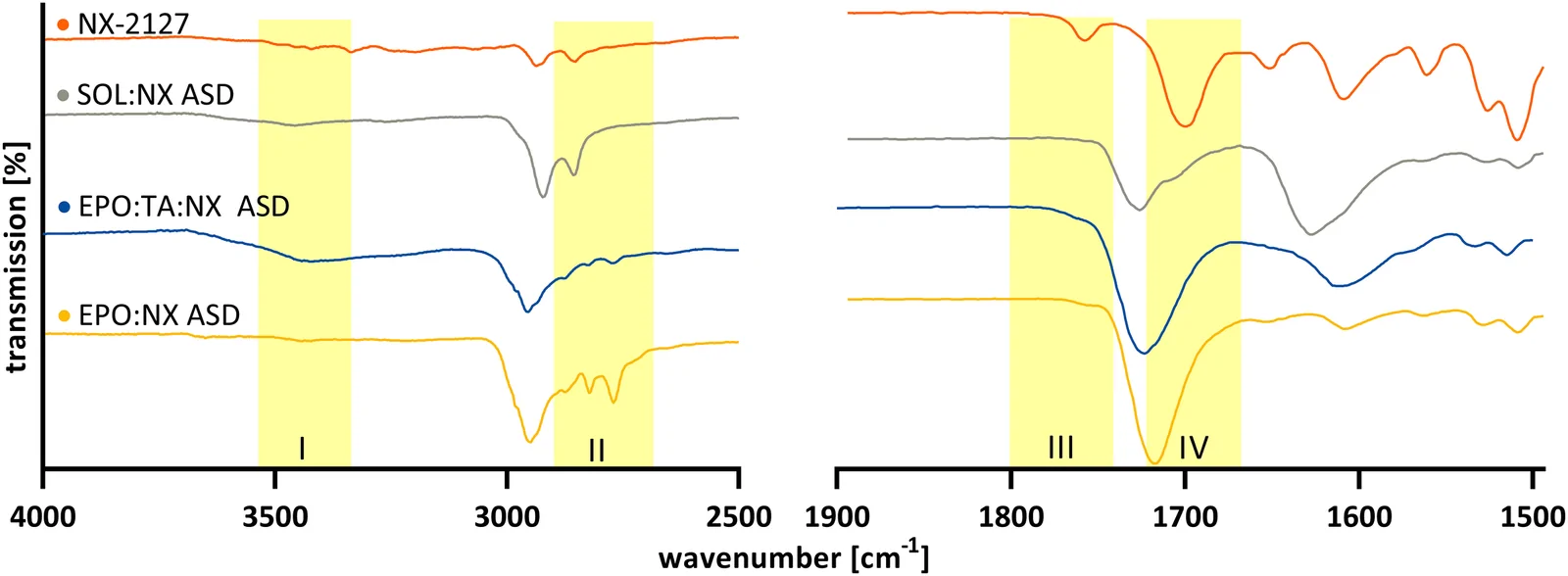
Fourier-transform infrared (FT-IR) spectra of  pure NX-2127 and 10% drug-loaded ASDs of  SOL (ASD1),  EPO (ASD2) and  EPO-tartaric acid (ASD3). Representative spectra are shown (*n* = 3): 4000 cm^−1^-2500 cm^−1^ and 1900 cm^−1^ -1500 cm^−1^. Yellow regions (I-IV) highlight the spectral areas discussed in the text.

The resulting Fourier-transform infrared (FT-IR) spectra of pure drug substance and the produced amorphous solid dispersions are given in Fig. 9. To highlight the decisive areas of the spectrum visible, the axis was divided, displaying only the ranges from 4000 to 2500 cm^−1^ and 1900 to 1500 cm^−1^. There were no visible peaks between 2500 and 1900 cm^−1^. The fingerprint range below 1500 cm^−1^ was excluded to simplify the figure. Two characteristic and distinct peaks (Fig. 9: III and IV) at 1763 cm^−1^ and 1706 cm^−1^ were observed for the raw drug substance NX-2127. These can be assigned to the symmetric and asymmetric C=O stretching vibrations typical of imide structures. The corresponding imide NH stretching vibration is only slightly visible at 3335 cm^−1^ (Küster and Thiel, 2023). Interestingly, the peaks associated with the imide structure (Fig. 9: III and IV) disappeared upon the formation of the ASD in all cases. This effect is particularly evident for the band at 1763 cm^−1^ (Fig. 9: III), which is still present in the physical mixture but is absent in the ASD (Supplementary Data, Fig. S6). Both SOL and EPO exhibit carbonyl bands due to their chemical structures, appearing at 1732 cm^−1^ for SOL and 1724 cm^−1^ for EPO, respectively. This overlap complicates the distinction of band IV at 1706 cm^−1^ (Fig. 9: IV), which is characteristic of the PROTAC's imide, from the polymer's carbonyl vibrations. Nevertheless, the complete disappearance of band III at 1763 cm^−1^ (Fig. 9: III) suggests an interaction between the imide group of the PROTAC and the polymer.

Additionally, the region at higher wavenumbers is of particular interest in the case of Eudragit® EPO. For ASD 2, which consisted of 90% EPO and 10% NX-2127, the characteristic bands of EPO's non-ionized dimethylamino group can were observed at 2821 cm^−1^ and 2769 cm^−1^ (Fig. 9: II). These bands were considerably smaller in the case of ternary ASD 3, for which EPO was partially protonated through the addition of TA. This can be confirmed by the broad NH stretching vibration occurring at 3418 cm^−1^ (Fig. 9: I), which is characteristic for the quaternary nitrogen of EPO after processing (Mild et al., 2026; Jeganathan and Prakya, 2015; Fan et al., 2021).

To gain mechanistic insights into the stabilization of supersaturation in aqueous solution by the investigated polymers, principal component analysis (PCA) and partial least squares (PLS) regression were conducted. PCA revealed that the second principal component was primarily driven by descriptors related to carbonyl- and ester-containing functionalities (fr_C_O_noCOO, fr_ester), charge distribution (PEOE_VSA6, MaxPartialCharge, MinAbsPartialCharge), and electronic surface properties (VSA_Estate2, Estate_VSA8, VSA_Estate8, SMR_VSA10) (Fig. S7, Table S3). The first two principal components explained 63.0% of the total variance. These descriptors indicate that molecular polarity and the presence of electronically active surface regions are major factors contributing to the variation within the polymer dataset. To further investigate the descriptor-precipitation inhibition relationships of the polymers, PLS regression was performed. The descriptors with the highest variable importance plots (VIP) included fr_ester, fr_C_O_noCOO, PEOE_VSA6, PEOE_VSA3, EState_VSA8, VSA_EState4, MolLogP, SlogP_VSA6, HallKierAlpha, and MinAbsEStateIndex (Fig. S8, S9). Notably, several influential descriptors identified by PLS overlapped with those highlighted by PCA, particularly descriptors related to ester and carbonyl functionalities as well as electronic surface characteristics. Overall, both multivariate analyses consistently suggest that polymer-NX-2127 interactions in aqueous solutions are strongly associated with the abundance of polar oxygen-containing functional groups, distribution of electronic charge across the molecular surface, and accessibility of electronically active regions. In addition, lipophilicity-related descriptors contributed to the observed variation, indicating that both polar and hydrophobic interactions may influence the interaction behavior of the investigated polymers with the PROTAC. Although PCA and PLS do not directly reveal the underlying molecular mechanism, the importance of these descriptors suggests that hydrogen-bonding and dipole-dipole interactions are likely key contributors to polymer performance as precipitation inhibitors. Such interactions may promote polymer-drug association in solution, thereby contributing to the maintenance of supersaturation.

### 3.4 Non-sink dissolution study

**Fig. 10:**
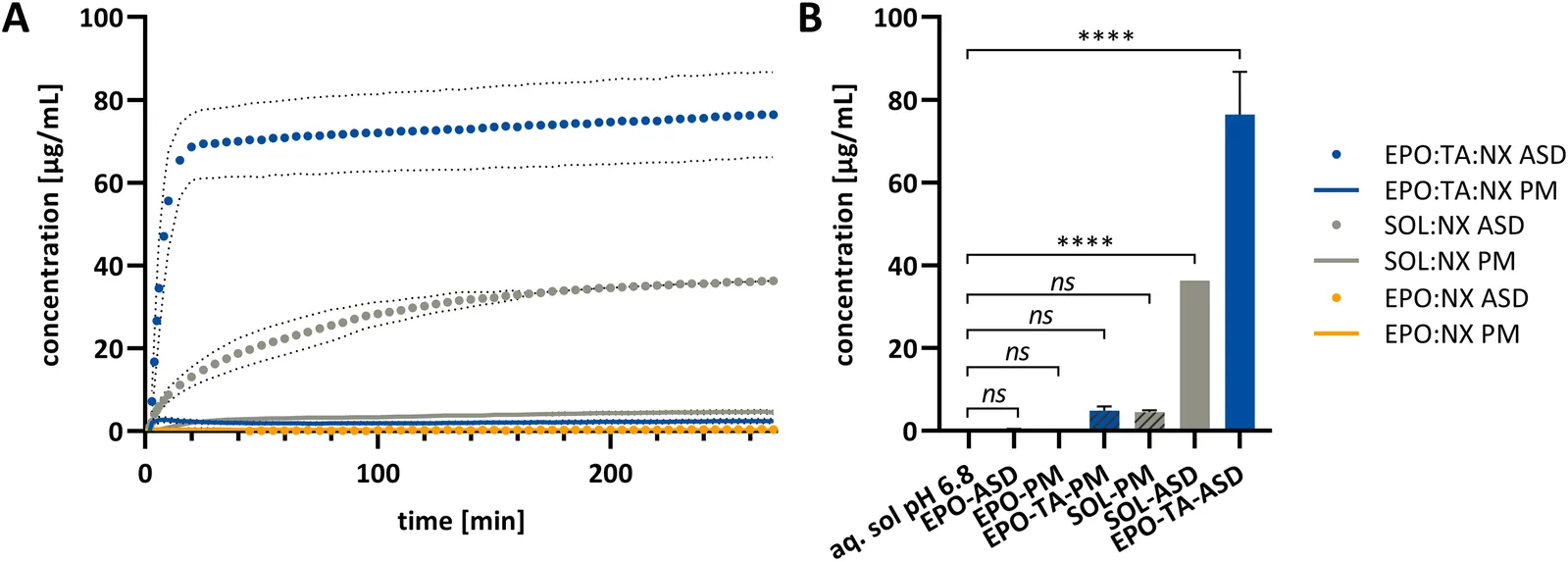
A: Non-sink dissolution profile of the following ASD and PM with a drug load of 10% ( SOL PM 1, ASD 1,  EPO PM 2, ASD 2 and  EPO-tartaric acid PM 3, ASD 3) in 20 mL of 0.05 M phosphate buffer adjusted to pH 6.8 (37 °C, 75 rpm paddle speed, 270 min). Data are presented as mean ± SD (*n* = 3). B: Endpoint concentrations after 270 min. Bars represent the mean ± SD (*n* = 3). Statistical analysis was performed using one-way ANOVA followed by Dunnett's multiple comparisons test *vs.* aqueous solubility at pH 6.8: * *p* < 0.05, ** *p* < 0.01, *** *p* < 0.001, **** *p* < 0.0001.

Dissolution experiments were conducted under strict non-sink conditions in phosphate buffer pH 6.8 with an sink index (SI) calculated according to Sun et al. (Sun et al., 2016) of 0.0007. The physical mixtures of all the examined polymers did not demonstrate any significant enhancement in dissolution (*p* > 0.05, Fig. 10B). In contrast, the ASD, composed of SOL and NX-2127, exhibited the capacity to stabilize a supersaturated solution up to 36.27 ± 0.02 μg/mL after 270 min (Fig. 10A). Contrary to previous expectations, the ASD with NX-2127 and EPO barely achieved supersaturation. A mere concentration of 0.40 ± 0.07 μg/mL was achieved (*p* > 0.05). Given that EPO only dissolves below a pH of 5, the dissolution was likely limited due to the carrier's poor solubility in the experimental setup. Consequently, 20% TA was incorporated during the vacuum compression molding process to protonate the tertiary nitrogen within the dimethylamino side chain of EPO. As Mild et al. previously outlined, protonation-driven polymer solubilization occurs rapidly during dissolution, thereby enhancing drug release (Mild et al., 2026). This phenomenon can be similarly observed in the NX-2127 study, wherein the incorporation of 20% TA into the EPO matrix during VCM processing led to a significantly enhanced release of 76.45 ± 10.29 μg/mL (*p* < 0.0001, Fig. 10B).

### 3.5 Biphasic dissolution (BiPHa+)

**Fig. 11:**
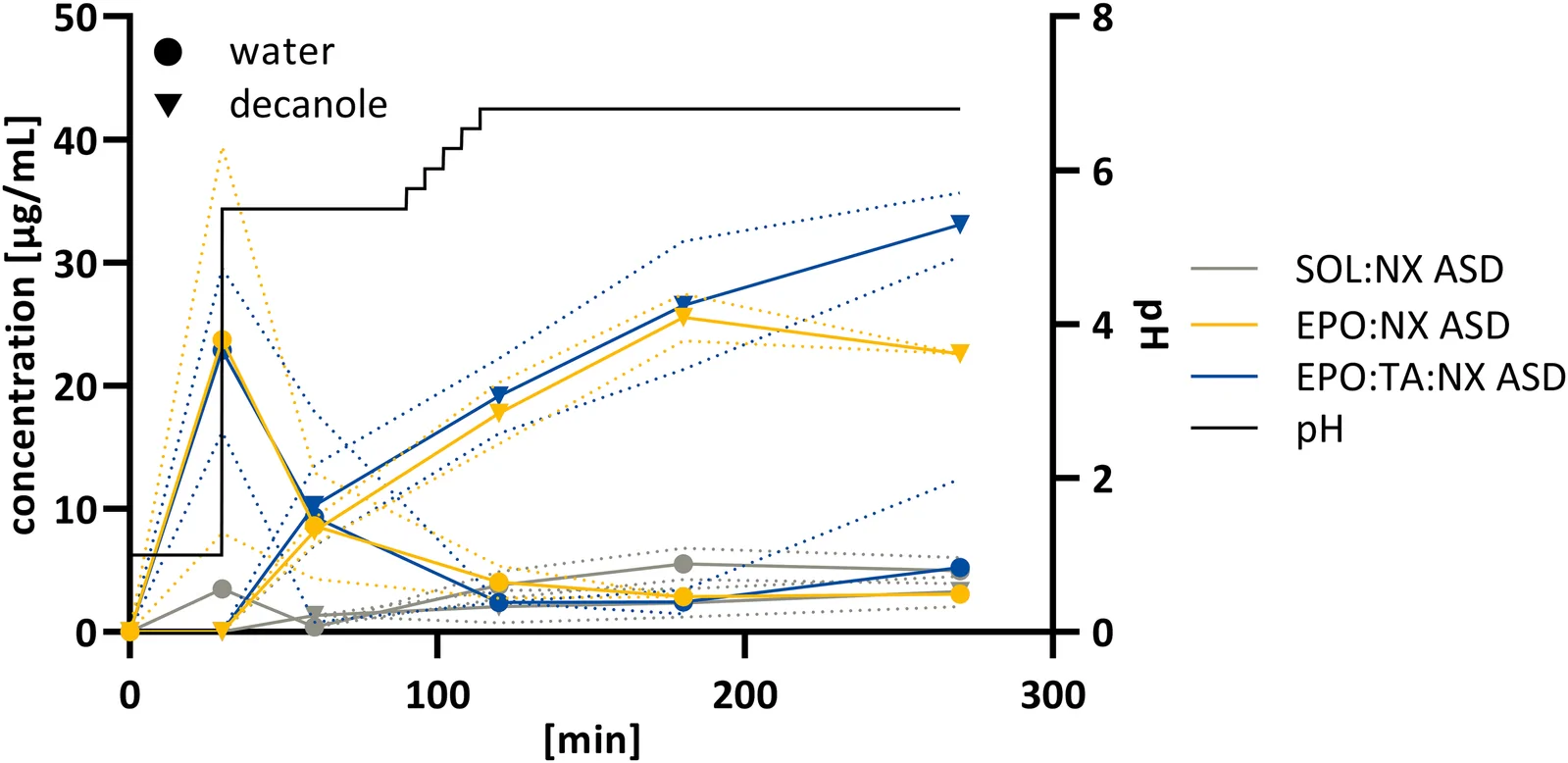
Dissolved and partitioned proportions of NX-2127 in the aqueous (•) and decanol (▼) phases, during the BiPHa+ dissolution study from the corresponding formulations:  ASD 1 (10% NX-2127, SOL-ASD),  ASD 2 (10% NX-2127, EPO-ASD) and  ASD 3 (10% NX-2127, EPO-tartaric acid-ASD). Values represented as mean ± SD (*n* = 3).

To evaluate the impact of the different ASD formulations on drug release and subsequent membrane permeation, the dissolution-permeation behavior of SOL- and EPO-based systems was investigated under biorelevant conditions (Fig. 11). Notably, only a concentration of 5.00 ± 0.13 μg/mL was released from the SOL-ASD into the aqueous phase after 30 min. After 270 min, only 3.29 ± 0.32 μg/mL had partitioned into the organic phase, indicating limited permeability of NX-2127 when incorporated into SOL micelles and reduced transfer into the decanol layer, which serves as a surrogate for the absorption compartment (Alopaeus et al., 2019; Pignatello et al., 2022).

In contrast, the EPO-based ASD showed a substantially improved performance. Overall, both formulations exhibited comparable dissolution profiles, characterized by an initial pronounced spring effect under acidic conditions, followed by a decrease in the dissolved NX-2127 concentration after the pH shift, simulating intestinal transit. However, an increased transfer into the decanol phase was observed at later time points in the absence and presence of TA. For the binary EPO-ASD, only 22.57 ± 0.04 μg/mL diffused into the upper, organic phase. The incorporation of TA into the EPO-ASD further enhanced this effect by approximately 50%, resulting in 33.09 ± 2.58 μg/mL after 270 min.

## 4 Discussion

NX-2127 is a basic compound with a predicted pK_a_ of 8.38, as calculated using the ADMET Predictor (Table 1). Consistent with its basic nature, the compound exhibited pronounced pH-dependent thermodynamic solubility. Additionally, a 49-fold increase in thermodynamic solubility under fed-state conditions highlighted a substantial food effect, a phenomenon commonly reported for PROTACs (Srivastava et al., 2025; Pike et al., 2020; He et al., 2024). In line with this observation, He et al. demonstrated increased oral BA of ARV-110 in rats under fed conditions, with BA increasing from 10.75% to 20.97% (He et al., 2024). Together, these findings suggest that food intake markedly enhances the solubilization and absorption of such highly lipophilic and structurally complex molecules. To evaluate the solubility of NX-2127 while minimizing compound consumption, both *in silico* prediction and experimental second harmonic scattering (SHS) approaches were applied. The ADMET Predictor substantially overestimated the water solubility, predicting a value of 64 μg/mL for the compound. This discrepancy underscores the limitations of commonly used predictive models in accurately estimating the thermodynamic solubility of complex PROTAC molecules (Poongavanam et al., 2023; García Jiménez et al., 2022). Conversely, the SHS method required only minimal amounts of the active ingredient and provided results comparable to those obtained by conventional LC-MS analysis, while relying on a technically less demanding setup (Kalasová et al., 2025). Using SHS, the solubility of NX-2127 was determined to be 0.122 ± 0.05 μg/mL, which closely matched the value obtained by LC-MS analysis (0.140 ± 0.057 μg/mL). Solid-state characterization further revealed that NX-2127 was fully amorphous, as confirmed by DSC and XRPD analyses. This observation is consistent with literature reports describing PROTAC molecules as generally poor crystallizers due to their high molecular complexity and conformational flexibility (Pöstges et al., 2023; Screen et al., 2025b; Torodii et al., 2025).

Based on these physicochemical properties, the ASD approach was selected as the main formulation strategy to enhance the apparent solubility and dissolution behavior of NX-2127. To minimize API consumption during formulation development, a combined *in silico* and *in vitro* screening strategy was implemented to identify suitable polymeric carriers. The most promising candidates were subsequently evaluated for their capacity to form a physically stable molecular amorphous dispersion in solid state as well as to achieve and maintain drug supersaturation. COSMOquick predictions identified HPMCAS, EPO and L100–55 as the three most promising precipitation inhibitors, whereas SOL was ranked only fifth. However, because the underlying COSMO-RS model does not explicitly consider water as an interacting component, its predictive capability for the induction and maintenance of supersaturation in aqueous, biorelevant media is inherently limited, especially in the case of polymers with limited hydration. Subsequent supersaturation screening experiments revealed that EPO and SOL were particularly effective in generating and stabilizing supersaturation. Furthermore, HPMC, PVA and MC exhibited precipitation-inhibiting properties once a metastable supersaturated state had been established. In contrast to the *in silico* predictions, the polymers HPMCAS and L100–55 did not demonstrate a pronounced time-dependent stabilizing effect on maintenance of supersaturation in the liquid state, further highlighting the limitations of the computational model for accurately describing complex drug-polymer interactions in solution. Although COSMOquick proved useful for the initial assessment of polymer suitability, the partial discrepancies observed between the predicted and experimental performance demonstrate that it should primarily be regarded as a supportive pre-screening tool. Consequently, experimental *in vitro* screening remains essential for reliable polymer selection.

A more detailed evaluation of the supersaturation kinetics revealed that, contrary to the initial assumption that high C_max_ levels initiate immediately at the addition of NX-2127 DMSO stock solution, followed by a gradual precipitation, the amount of supersaturated NX-2127 in solution accumulated over time from dissolving precipitates. The polymers were able to stabilize this meta-stable state of redissolved precipitates over prolonged periods, resulting in a dissolution-like profile. Simultaneously, the precipitated fraction formed immediately after the addition of NX-2127 gradually decreased in particle size over the course of the experiment. For SOL, in particular, particles with sizes of approximately 100 nm were detectable after 45 min. This observation may indicate the formation of micellar structures into which the PROTAC subsequently partitions. Such behavior could explain the atypical supersaturation profiles observed during the assay (Fig. S2). Overall, these findings further demonstrate that the predictive capability of the COSMOquick screening method is limited when translated into real solvent environments, especially for structurally complex and highly flexible molecules such as PROTACs, as previously discussed in the literature (Price et al., 2019; Cordova et al., 2024; Jha et al., 2025). Additionally, the COSMOquick calculation does not capture the formation of micelles or colloidal structures. Nevertheless, the general trends predicted *in silico* showed partial agreement with the experimental *in vitro* results. In summary, the combined screening strategy enabled a substantial reduction in the number of polymer candidates prior to ASD preparation, thereby streamlining subsequent formulation development while minimizing API consumption.

Monophasic dissolution testing at pH 6.8 further differentiated the polymeric systems. SOL-based ASDs demonstrated superior dissolution performance compared to the binary EPO formulation, as EPO does not dissolve at pH levels above 5.0. However, as previously introduced by Mild et al. (Mild et al., 2026), the incorporation of an organic acid into an EPO matrix during processing can enhance dissolution, independent of pH. This platform was implemented following the integration of tartaric acid (TA) into the binary ASD comprising EPO and NX-2127. Consequently, a 546-fold increase in the apparent NX-2127 solubility was observed. The addition of TA led to protonation of the tertiary amine group in the polymer backbone, thereby increasing the solubility of the EPO matrix itself, especially at pH values above 5.0. As EPO is usually only soluble below pH 5.0, this protonation notably accelerates polymer dissolution and consequently the release of NX-2127. Mild et al. (Mild et al., 2026) offered a more in-depth examination of the underlying mechanisms, polymer and acid stability, as well as interactions. FT-IR analysis has further indicated specific interactions between the imide moiety of NX-2127 and the polymers EPO and SOL. The characteristic bands associated with the imide functional group disappeared upon processing into the ASD, suggesting the formation of intermolecular interactions in the solid state. As reported in the literature, such API-polymer interactions can notably influence both the thermodynamic and kinetic behavior of supersaturated systems, thereby enhancing drug stabilization in solution. This effect is especially pronounced when hydrogen bonding is involved. In the present case, hydrogen bond formation may occur between the imide hydrogen atom of NX-2127 and the functional side chains of EPO and SOL, which could contribute to the observed stabilization of supersaturation and improved dissolution behavior (Qin et al., 2022; Baghel et al., 2016; Prasad et al., 2016). This interpretation is further supported by the PCA and PLS regression results, which identified descriptors associated with carbonyl and ester functionalities as well as electronic surface properties as the main contributors to the structural variation within the polymer dataset. The prominence of these descriptors suggests that the availability of hydrogen-bond acceptor groups and favorable charge distributions are important factors governing polymer performance. Consequently, both hydrogen-bonding and dipole–dipole interactions are likely involved in the polymer-mediated stabilization of supersaturation in aqueous media.

In order to reflect pH change and potential passive absorption mechanisms, further evaluation in the BiPHa+ system was conducted (Denninger et al., 2020; Denninger et al., 2021). Differences in drug dissolution and distribution behavior between the formulations were revealed. The SOL-ASD showed limited distribution of solubilized NX-2127 into the decanol layer. This effect is likely due to the micelle-forming properties of SOL, as we exceeded the critical micelle concentration of 0.5 mg/mL in MilliQ water (Alopaeus et al., 2019; Pignatello et al., 2022). The resulting micelles, with sizes below 100 nm (Fig. S2), are unlikely to efficiently cross the interface between aqueous medium and the decanol phase due to their limited molecular mobility. Furthermore, NX-2127 may remain entrapped within the micellar structures without undergoing sufficient release, thereby restricting its distribution into the organic phase. (Alopaeus et al., 2019; Pignatello et al., 2022; Mateos et al., 2022). In contrast, the effect of acid incorporation in EPO-based ASDs was less pronounced at low pH, since polymer solubility is already sufficient at pH 1. However, after the pH shift to 6.8, clear differences were observed. For the binary EPO-ASD, partition of NX-2127 in the decanol layer decreased after approximately 180 min when reaching a pH of 6.8 in the aqueous phase. This is possibly due to the poor solubility and fast precipitation of EPO itself at this pH (Mild et al., 2026). In contrast, no such decrease was observed when acid was incorporated into the EPO matrix. The presence of the organic acid-maintained polymer solubility after the pH shift, resulting in sustained supersaturation of NX-2127 and a predicted 50% increase in drug absorption in the BiPHa+ system compared to that in the binary EPO-ASD (from 22 μg/mL to 33 μg/mL). Overall, the developed ASD formulation successfully addressed the major limitations of NX-2127 by enhancing apparent solubility, enabling sustained supersaturation, and providing a promising strategy to improve the oral performance of this challenging PROTAC molecule.

## 5 Conclusion

In conclusion, this work demonstrated that the challenging properties of NX-2127 can be effectively addressed through the development of an amorphous solid dispersion (ASD) formulation. The compound exhibited extremely low aqueous solubility, pronounced pH dependency, and a strong food effect, all of which are characteristic limitations of PROTAC molecules. By combining *in silico* and *in vitro* screening approaches, suitable polymeric carriers were efficiently identified while minimizing API consumption during early-stage formulation development. Although the predictive capability of the COSMOquick approach was limited for accurately describing supersaturation behavior in complex solvent environments, the combined screening strategy successfully identified EPO and SOL as particularly promising precipitation inhibitors. These polymers enabled the generation and long-term stabilization of supersaturated states, resulting in improved dissolution behavior, likely due to favorable hydrogen-bonding and dipole-dipole interactions arising from their polar functional groups and electronic surface characteristics.

Overall, the developed ASD formulation represents a promising strategy to overcome key formulation challenges associated with PROTACs and provides valuable insights into the interplay between polymer selection, supersaturation behavior, and molecular interactions in these highly complex drug candidates. However, it is important to emphasize that these promising results need to be supported by further *in vivo* studies.

## Declaration of generative AI and AI-assisted technologies in the manuscript preparation process

During the preparation of this manuscript, the author used ChatGPT and DeepL Write to support language editing and to improve the clarity of the abstract, title, graphical abstract, and highlights. All generated suggestions were carefully reviewed and revised where necessary, and the author assumes full responsibility for the final content of the published article.

## CRediT authorship contribution statement

**Alicia Stakemeier:** Writing – original draft, Visualization, Methodology, Investigation, Formal analysis, Data curation, Conceptualization. **Lukas Mild:** Writing – review & editing, Visualization, Methodology, Investigation. **Johannes Sievers:** Methodology, Investigation. **Jitka Kalasová:** Writing – review & editing, Visualization, Methodology, Investigation. **Kristina E. Steffens:** Writing – review & editing, Methodology, Investigation. **Martin Kuentz:** Writing – review & editing, Methodology, Investigation. **Christian Steinebach:** Writing – review & editing, Supervision, Resources, Methodology, Investigation. **Karl G. Wagner:** Writing – review & editing, Supervision, Resources, Methodology, Funding acquisition, Conceptualization.

## Ethics declaration

The author(s) declare(s) that the study does not involve humans nor animal subjects.

## Funding

This research did not receive any specific grant from funding agencies in the public, commercial, or not-for-profit sectors.

## Declaration of competing interest

The authors declare that they have no known competing financial interests or personal relationships that could have appeared to influence the work reported in this paper.

## Data Availability

Data will be made available on request.
