# Supplementary material for: From prediction to performance: In silico-supported formulation of amorphous solid dispersions for PROTAC NX-2127
Source: Int J Pharm X. 2026 Sep 10;12:100663. doi: 10.1016/j.ijpx.2026.100663 (PMC13594704; doi:10.1016/j.ijpx.2026.100663)
Supplement: Supplementary file 1 [file mmc1.pdf]

Supplementary

METHODS

LC-MS

Compound purity and identity were determined by HPLC-UV using an LC-MS instrument (Agilent Infinity Lab LC/MSD-system with ESI source coupled with an Agilent HPLC 1260 Infinity II). For LC/MS analysis, an EC50/2 Nucleodur C18 Gravity 3 µm column (Macherey-Nagel) was used. The column temperature was maintained at 40 °C. HPLC separation started with 90% H_2O containing 2 mM ammonium acetate. The gradient was ramped to 100% acetonitrile over 10 min, followed by flushing with 100% acetonitrile for 5 min. The flow rate was 0.5 mL/min. Samples were dissolved in water, methanol, or acetonitrile (≈1 mg/mL), and 2 µL of the sample solution was injected. Positive total ion scans were recorded from 100 to 1000 *m*/*z* (or higher if necessary), and UV absorption was detected from 190 to 600 nm using a diode array detector (DAD). Purity was determined at 220–600 nm unless otherwise specified. All compounds that were evaluated were ≥95% pure by LC/MS analysis.

NMR spectroscopy

^{1}H NMR and ^{13}C NMR spectra were recorded on Bruker Avance 500 MHz or Bruker Avance III 600 MHz NMR spectrometers. NMR spectra were processed and analyzed using MestReNova software. Chemical shifts (δ) are reported in parts per million (ppm), coupling constants (*J*) are reported in hertz (Hz), and spin multiplicities are designated as s (singlet), d (doublet), t (triplet), q (quartet), or m (multiplet). In cases of overlapping extraneous solvent peaks, multiplet analyses in ^{1}H NMR spectra were performed using quantitative Global Spectral Deconvolution (qGSD).

FIGURES

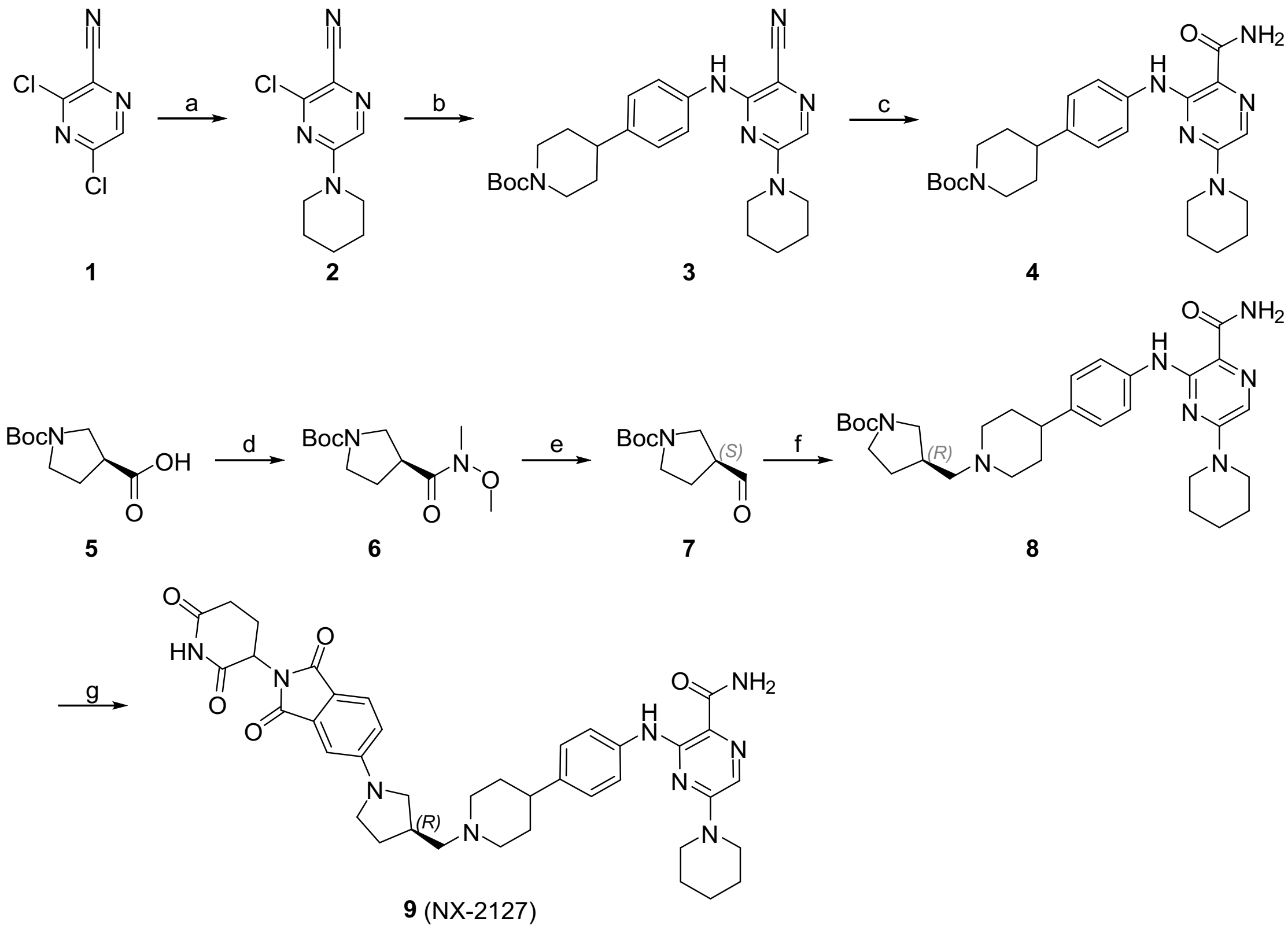

Figure S1: Gramm-scale synthesis of NX-2127. *Reagents and Conditions*: (a) Piperidine, DIPEA, DMF, rt, 2 h, 60%; (b) *tert*-butyl 4-(4-aminophenyl)piperidine-1-carboxylate, $Pd(OAc)_2$, Cs_2CO_3, BINAP, dioxane, 100 °C, 4 h, 71%; (c) H_2O_2, K_2CO_3, DMSO, rt, 24 h, 95%; (d) **5**, *N*,*O*-dimethylhydroxylamine × HCl, EDC × HCl, DMAP, DIPEA, dry DMF; rt, 18 h, 72%; (e) $LiAlH_4$, THF, 1 h, 0 °C, 80%; (f) (i) **4**, TFA, CH_2Cl_2, rt, 2 h; (ii) **7**, CH_2Cl_2, (iii) STAB, THF, rt, 18 h, 40%; (g) (i) **8**, TFA, CH_2Cl_2, rt, 2 h; (ii) 2-(2,6-dioxopiperidin-3-yl)-5-fluoroisoindoline-1,3-dione, DIPEA, DMSO, 90 °C, 18 h, 31%.

Further details on the synthesis can be found in the last section “Experimental Details.”

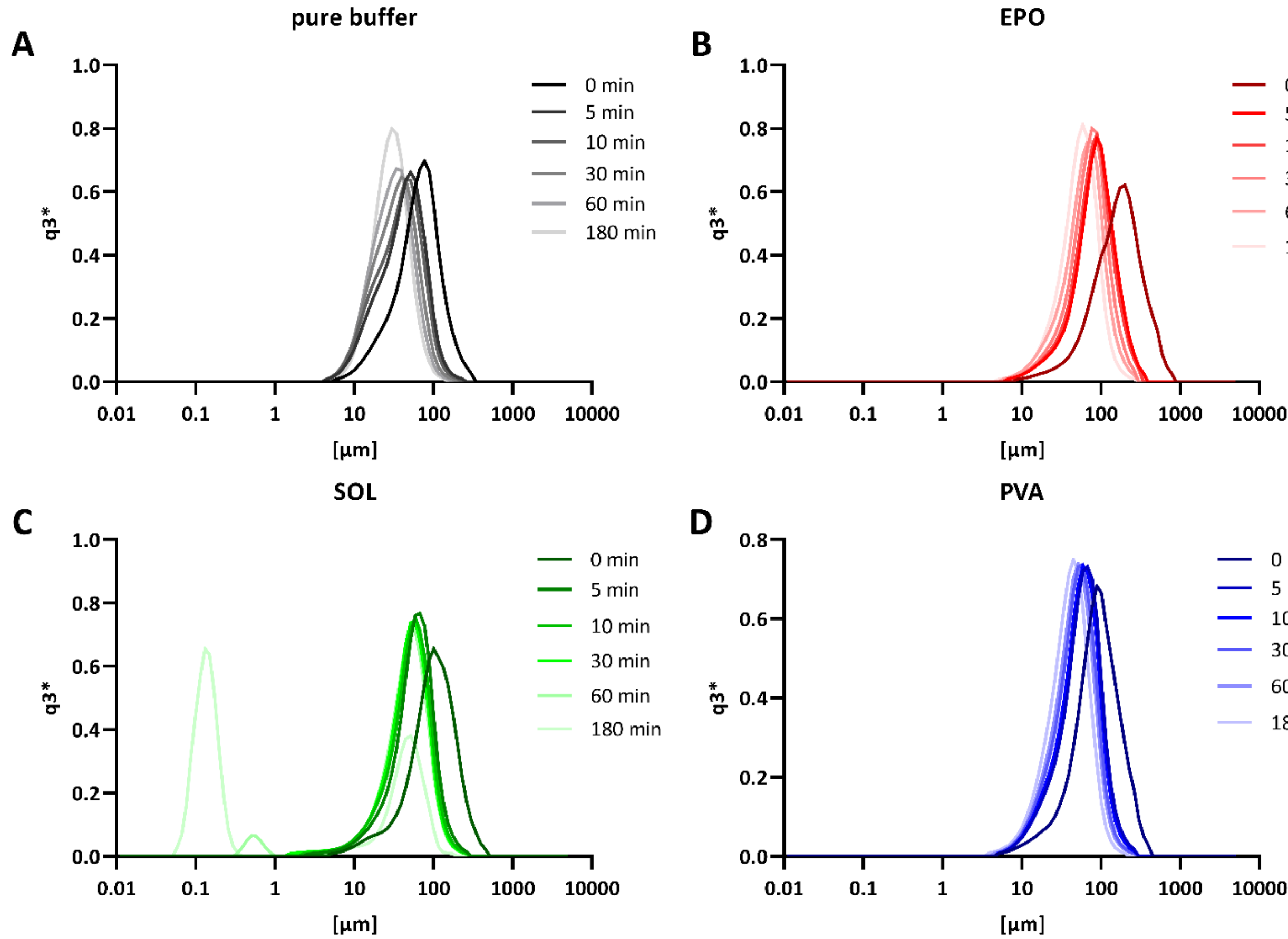

Figure S2: Volume-based particle size distributions (q3*) of precipitated NX-2127 measured by laser diffraction during dispersion in (A) 0.05 M phosphate buffer pH 6.8, (B) 1.25 mg/mL pre-dissolved EPO in 0.05 M phosphate buffer pH 6.8, (C) 1.25 mg/mL pre-dissolved SOL in 0.05 M phosphate buffer pH 6.8, (D) 1.25 mg/mL pre-dissolved PVA in 0.05 M phosphate buffer pH 6.8 at different time points (0-180 min). Values represent mean (n=3).

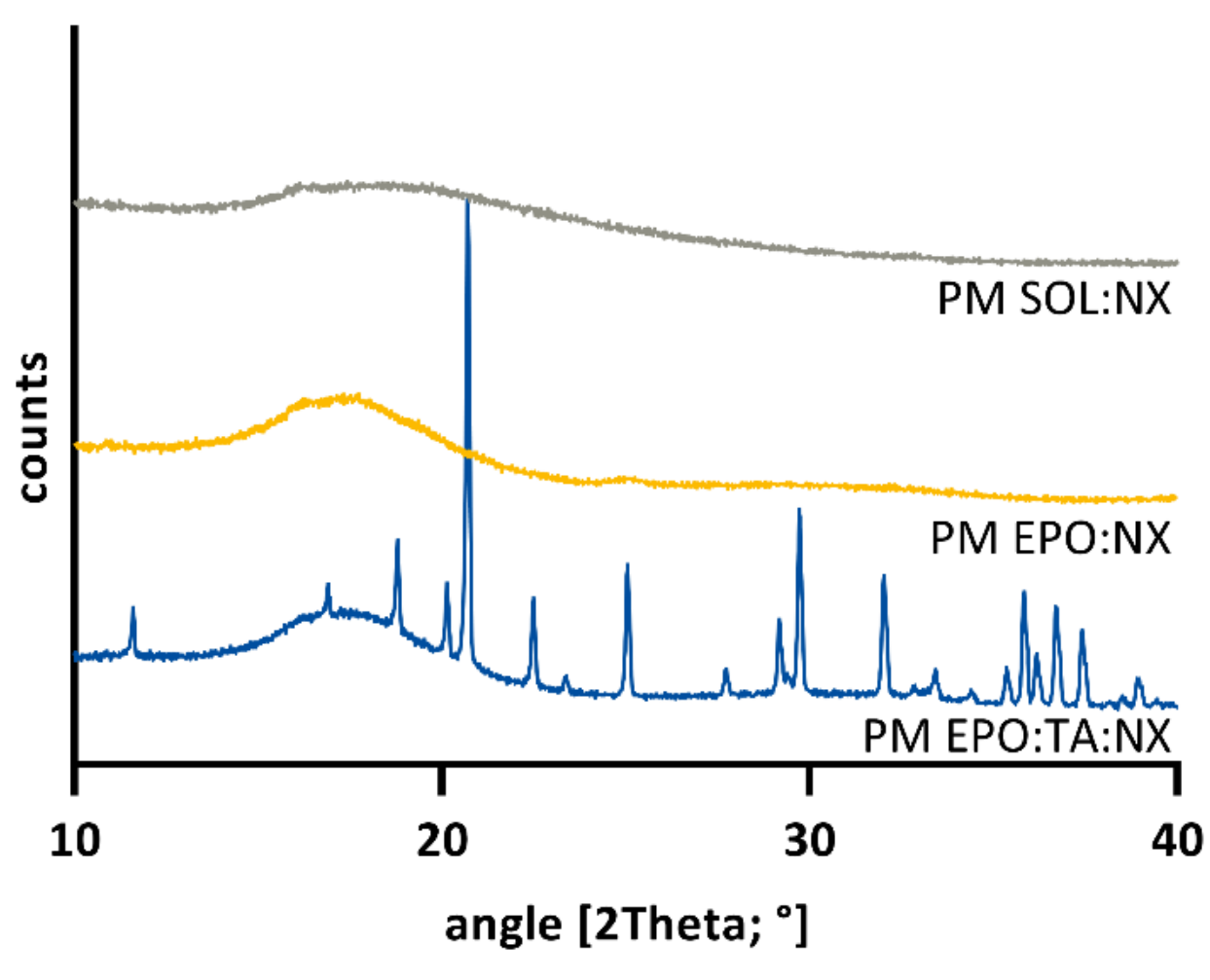

Figure S3: X-ray powder diffraction (XRPD) pattern 10% drug-loaded physical mixtures (PM): ● SOL (PM 1) and NX-2127, ● EPO (PM 2) and NX-2127 and ● EPO + TA (PM 3) measured in transmission mode with an angle from 10 – 40 ° 2θ. All samples were milled prior to analysis and measured in triplicate (*n* = 3). A representative spectrum is shown.

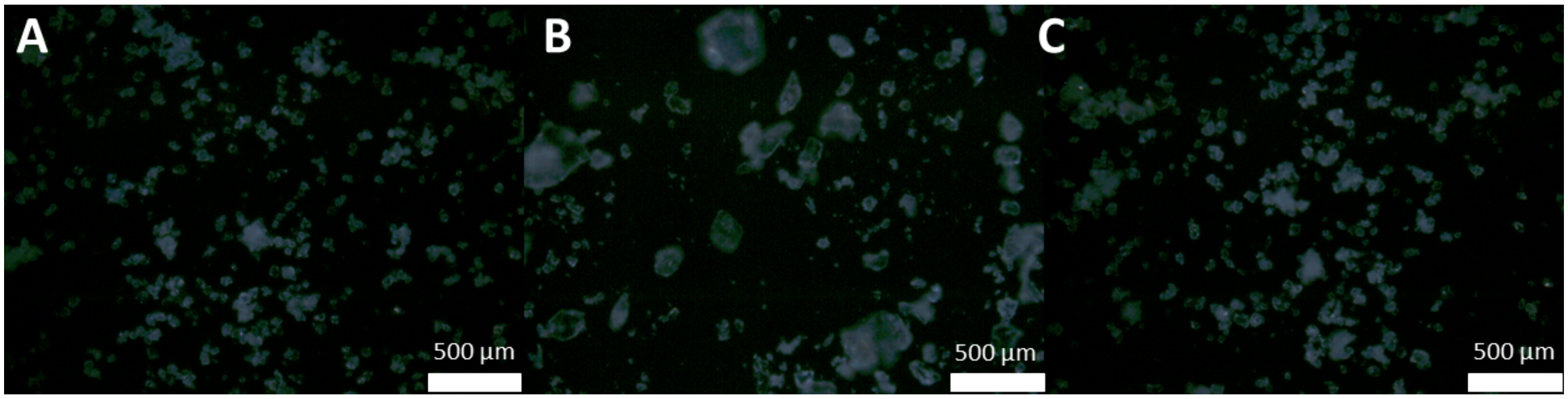

Figure S4: Polarized light microscopy images of 10% drug-loaded ASDS with different polymeric carriers: (A) EPO (B) EPO and TA (C) SOL. Representative images for each sample are shown.

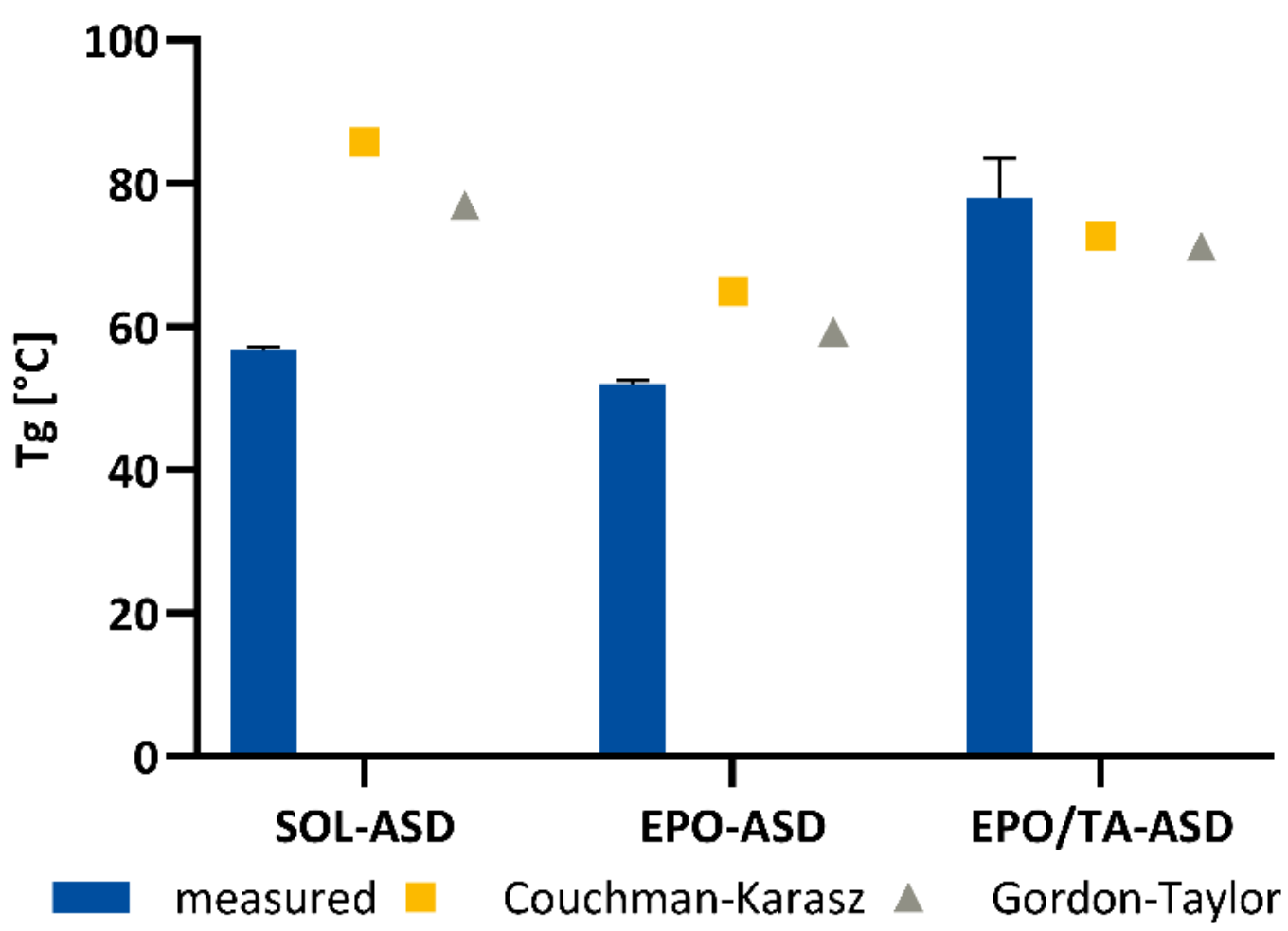

Figure S5: Residual plot of measured glass transition temperature (T*g*) of several ASDs compared to the predicted values according to Gordon-Taylor (GT) and Couchman-Karasz (CK) equations.

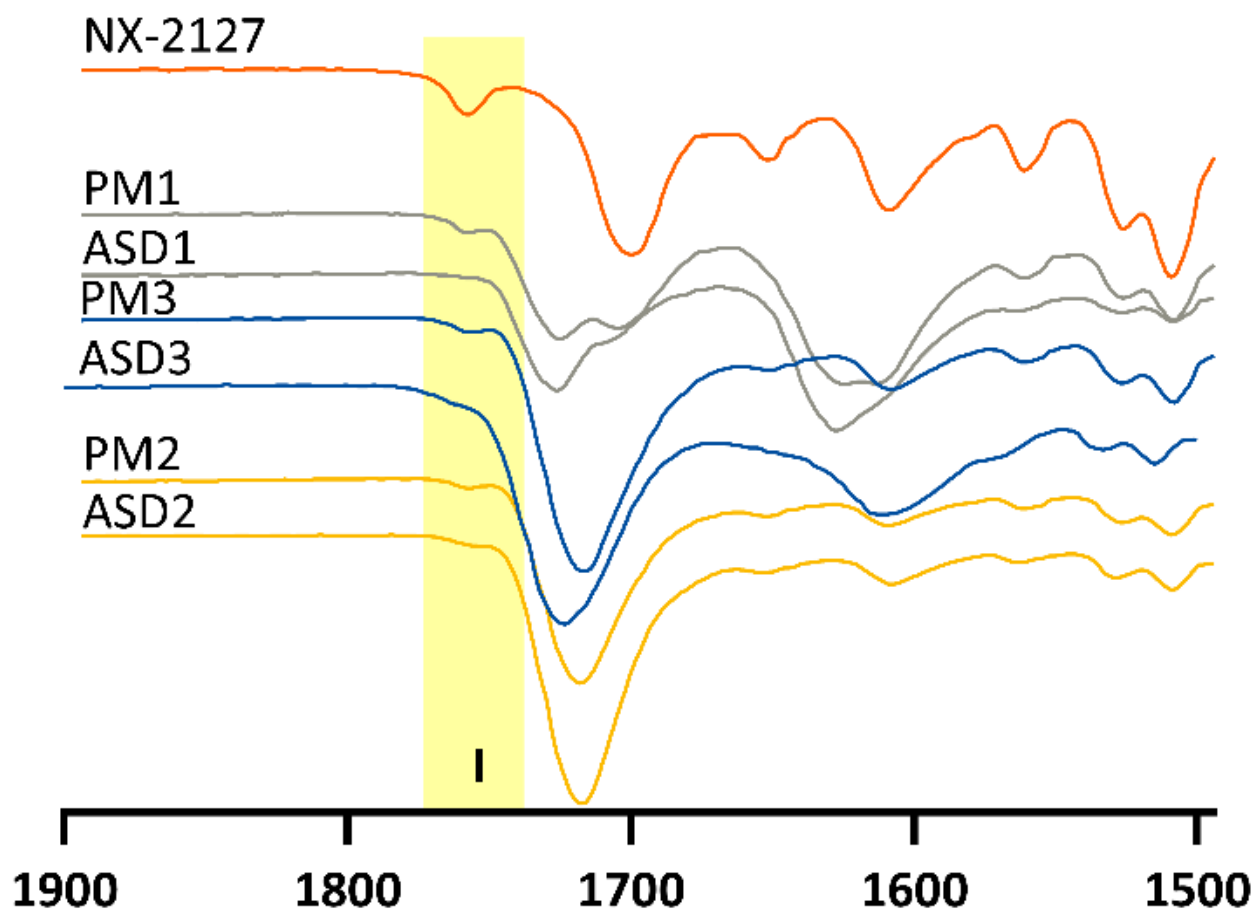

Figure S6: Fourier-transform infrared (FT-IR) spectra of • pure NX-2127 and 10% drug-loaded ASDs of • SOL (ASD1, PM1), • EPO (ASD2, PM2) and • EPO-tartaric acid (ASD3, PM3). Representative spectra are shown (n=3): 1900 cm^{-1} -1500 cm^{-1}. Yellow region (I) highlighting the spectral areas discussed.

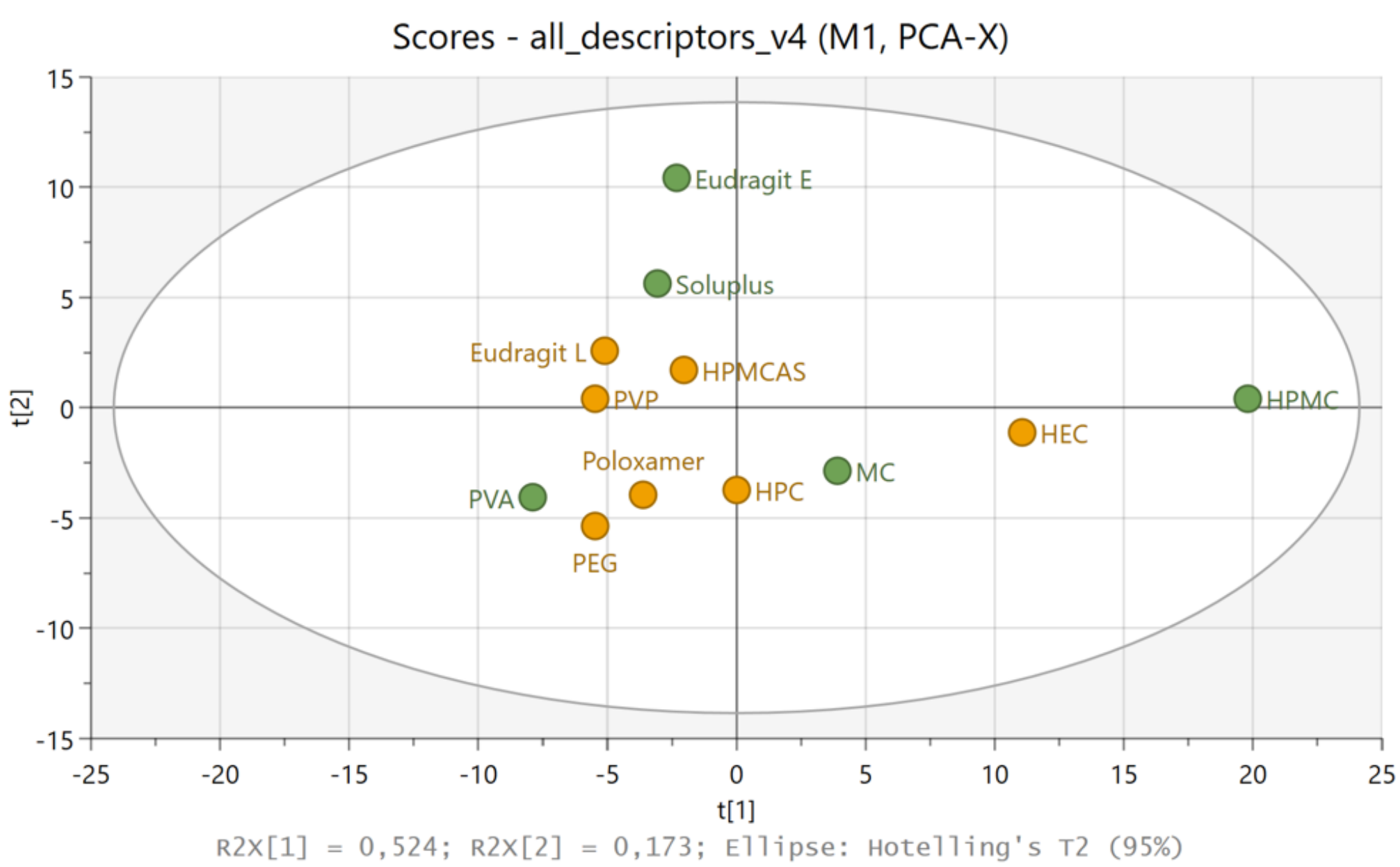

Figure S7: Score-Plot of Principal Component Analysis (PCA) of investigated polymers to identify molecular descriptors that best describing the polymer performance as precipitation inhibitor. green dots: precipitation inhibitors; orange dots: no precipitation inhibitors determined by supersaturation assay.

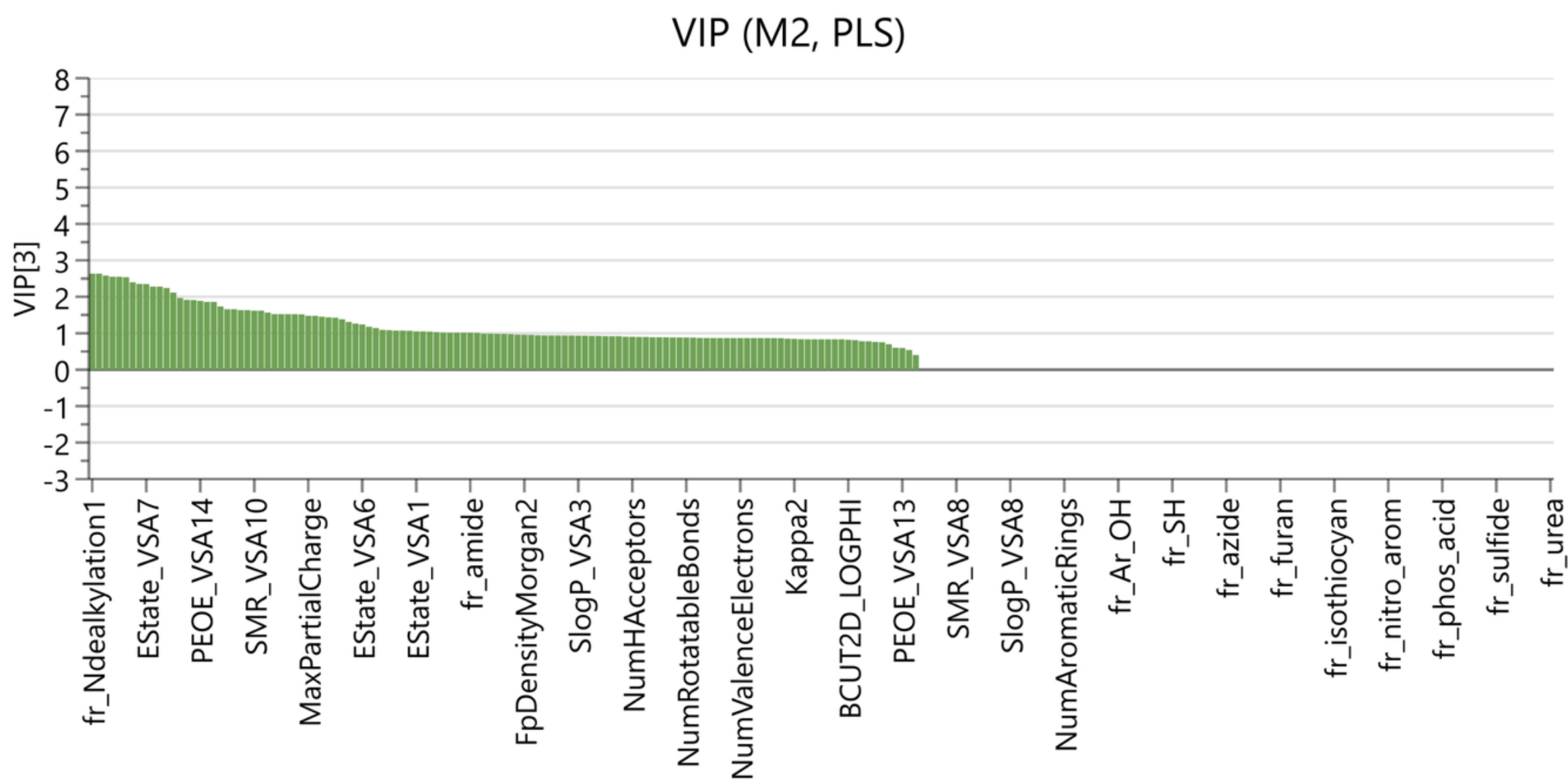

Figure S8: Variable importance plot (VIP) of PLS-regression. Top 10 VIP-values were discussed in the text.

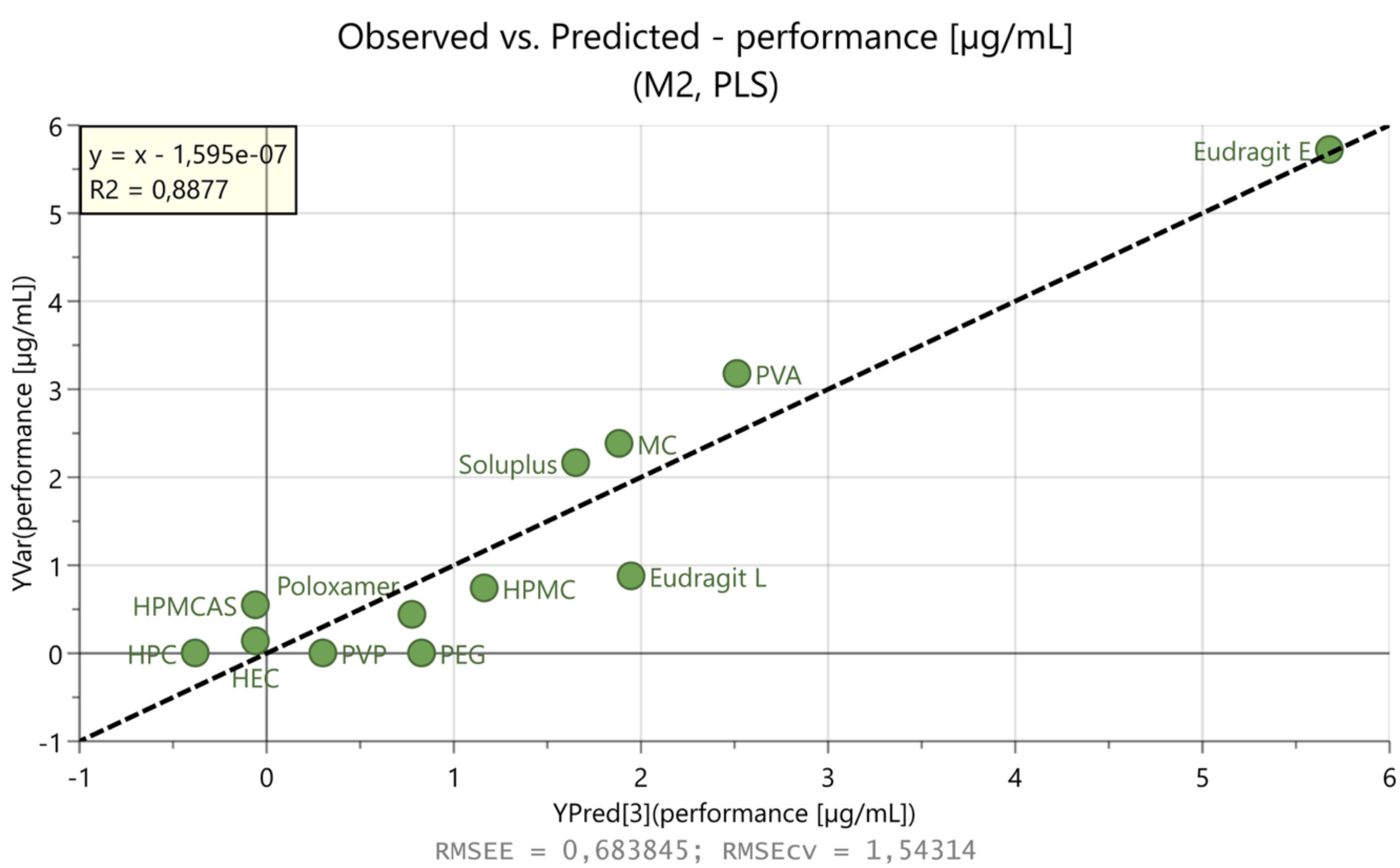

Figure S9: PLS-model performance: experimentally determined values of supersaturation assay were plotted against the predicted values based on the model.

TABLES

Table S1: composition of biorelevant media FaSSIF-V2 and FeSSIF-V1 according to Teleki et al. (77)

	FaSSIF-V2	FeSSIF-V1
Sodium taurocholate [mM]	3	15
Lecithin [mM]	0.2	3.75
Maleic Acid [mM]	19.12	-
Acetic Acid [mM]	-	144
Sodium [mM]	106	319
Chloride[mM]	69	203
pH	6.5	5
Hydrodynamic diameter [nm]	32	8

Table S2: d90-values of volume-based particle size distributions of precipitated NX-2127 measured by laser diffraction during dispersion in either pure buffer or a polymer-solution (1.25 mg/mL) in 0.05 M phosphate buffer pH 6.8 at different time points.

Time [min]	*Pure buffer*	*EPO*	*SOL*	*PVA*
**0**	126.97 µm	354.04 µm	196.18 µm	183.62 µm
**5**	79.66 µm	158.45 µm	100.54 µm	102.20 µm
**10**	73.22 µm	147.66 µm	92.08 µm	96.56 µm
**30**	63.74 µm	126.04 µm	86.97 µm	86.80 µm
**60**	56.76 µm	110.24 µm	85.22 µm	82.42 µm
**180**	50.91 µm	92.06 µm	60.89 µm	71.33 µm

Table S3: Loadings of Principal Component Analysis (PCA) of investigated polymers to identify molecular descriptors that best describing the polymer performance as precipitation inhibitor.

PC1		PC2	
**Descriptor**	**Loading**	**Descriptor**	**Loading**
SlogP_VSA2	0,124038003	fr_C_O_noCOO	0,194877997
NumHAcceptors	0,123810001	VSA_EState2	0,191762
PEOE_VSA1	0,123755001	PEOE_VSA6	0,188308001
NOCount	0,123199001	MaxPartialCharge	0,187759995
NumHeteroatoms	0,123199001	MinAbsPartialCharge	0,187759995
SMR_VSA1	0,122916996	fr_ester	0,186900005
SMR_VSA5	0,122827999	EState_VSA8	0,186755002
NumAtomStereoCenters	0,122487001	fr_C_O	0,183411002
Chi2n	0,122483	SMR_VSA10	0,183323994
Chi3n	0,122399002	VSA_EState8	0,182876006

EXPERIMENTAL DETAILS

3-Chloro-5-(piperidin-1-yl)pyrazine-2-carbonitrile (2)

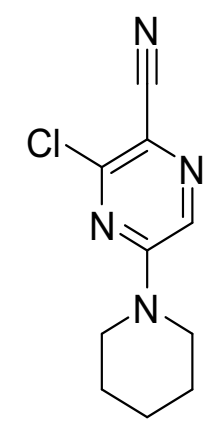

3,5-Dichloropyrazine-2-carbonitrile (**1**, 5.00 g, 28.7 mmol), piperidine (2.8 mL, 28.7 mmol), and DIPEA (6.0 mL, 34.5 mmol) were stirred in dry DMF for 3 h at rt. Then, 200 mL EtOAc was added, and the mixture was washed with saturated ammonium acetate (2 × 100 mL) and saturated sodium hydrogen carbonate solution (2 × 100 mL). The organic layer was dried over Na_2SO_4, filtered, and after the addition of Celite, concentrated *in vacuo*. Purification by flash chromatography on spherical silica gel (220 g, 30 µm, isocratic with dichloromethane) afforded the title compound as an off-white solid.

Yield (5.38 g, 84 %); R_f = 0.32 (20% EtOAc in petroleum ether); mp 79 – 82 °C; **^{1}H NMR** (500 MHz, DMSO-d_6) δ 1.54 – 1.61 (m, 4H), 1.61 – 1.69 (m, 2H), 3.69 – 3.75 (m, 4H), 8.38 (s, 1H); **^{13}C NMR** (126 MHz, DMSO-d_6) δ 23.77, 25.28, 45.48, 110.82, 116.41, 131.03, 149.33, 153.10; **LC-MS** (ESI) t_R = 6.90 min, 99 % purity, *m/z* $[M + H]^+$ calcd for $C_{10}H_{12}ClN_4$, 223.1; found, 223.1.

***Tert*-Butyl 4-(4-((3-cyano-6-(piperidin-1-yl)pyrazin-2-yl)amino)phenyl)piperidine-1-carboxylate (3)**

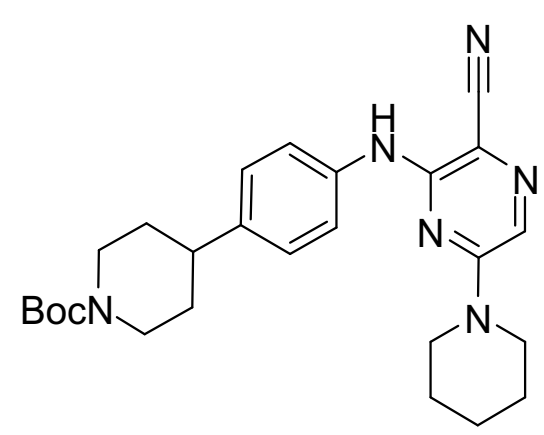

Compound **2** (5.34 g, 24.0 mmol), *tert*-butyl 4-(4-aminophenyl)piperidine-1-carboxylate (6.63 g, 24.0 mmol), $Pd(OAc)_2$ (1.08 g, 4.8 mmol), cesium carbonate (2.35 g, 72.0 mmol), and *rac*-BINAP (2.99 g, 4.8 mmol) were dissolved in dioxane before heating to 100 °C for 4 h. After cooling to room temperature, the mixture was diluted with 200 mL ethyl acetate and extracted with saturated sodium hydrogen carbonate solution (2 × 200 mL) and brine (200 mL). Purification by flash chromatography on spherical silica gel (220 g, 30 µm, gradient from 0 to 3 % MeOH in cyclohexane) afforded the title compound as an orange solid.

Yield (8.07 g, 73 %); R_f = 0.72 (20 % EtOAc in petroleum ether); mp 210 – 212 °C; **^{1}H NMR** (500 MHz, DMSO-d_6) δ 1.40 (s, 9H), 1.38 – 1.50 (m, 2H), 1.53 (td, *J* = 4.1, 6.8 Hz, 4H), 1.62 (q, *J* = 6.5 Hz, 2H), 1.69 – 1.77 (m, 2H), 2.62 (tt, *J* = 3.5, 11.9 Hz, 1H), 2.77 (s, 2H), 3.61 (dd, *J* = 4.3, 6.7 Hz, 4H), 4.05 (d, *J* = 13.0 Hz, 2H), 7.11 – 7.17 (m, 2H), 7.42 – 7.49 (m, 2H), 7.80 (s, 1H), 8.87 (s, 1H); **^{13}C NMR** (126 MHz, DMSO-d_6) δ 24.08, 25.32, 28.27, 33.01, 41.17, 45.14, 78.66, 98.62, 117.96, 121.31, 122.92, 126.59, 137.47, 140.36, 152.53, 152.75, 154.02; **LC-MS** (ESI) t_R = 9.26 min, 99 % purity, *m/z* $[M + H]^+$ calcd for $C_{26}H_{35}N_6O_2$, 463.3; found, 463.3.

***Tert*-Butyl 4-(4-((3-carbamoyl-6-(piperidin-1-yl)pyrazin-2-yl)amino)phenyl)piperidine-1-carboxylate (4)**

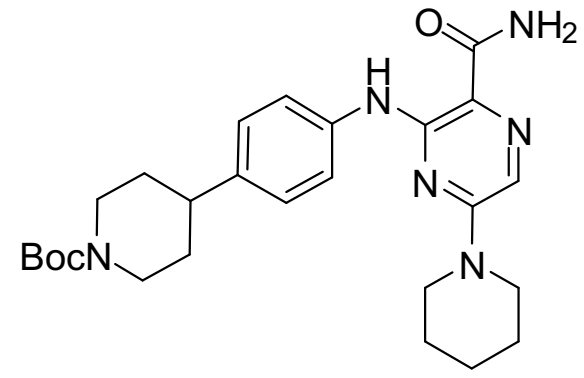

Compound **3** (7.86 g, 17.0 mmol) was dissolved in dry DMSO. Potassium carbonate (0.47 g, 3.4 mmol) and 35 % H_2O_2 solution (5.85 mL, 68.0 mmol) were added before stirring for 18 h at rt. The resulting mixture was diluted with 300 mL CH_2Cl_2 and extracted with brine (3 × 300 mL), dried over Na_2SO_4, and filtered through Celite. After evaporation *in vacuo*, a beige solid was obtained. This crude product was used without further purification.

Yield (7.76 g, 95 %); R_f = 0.70 (10 % MeOH in CH_2Cl_2); mp 195 – 197 °C; **^{1}H NMR** (600 MHz, DMSO-d_6) δ 1.40 (d, *J* = 1.7 Hz, 9H), 1.41 – 1.48 (m, 2H), 1.57 (h, *J* = 4.5 Hz, 4H), 1.61 – 1.75 (m, 4H), 2.61 (dd, *J* = 10.6, 13.7 Hz, 1H), 2.77 (s, 2H), 3.65 (t, *J* = 5.6 Hz, 4H), 4.05 (d, *J* = 11.3 Hz, 2H), 7.14 – 7.18 (m, 2H), 7.26 (d, *J* = 3.0 Hz, 1H), 7.48 – 7.53 (m, 2H), 7.64 (d, *J* = 1.7 Hz, 1H), 7.70 (d, *J* = 3.0 Hz, 1H), 11.24 (d, *J* = 2.2 Hz, 1H); **^{13}C NMR** (151 MHz, DMSO-d_6) δ 24.47, 25.58, 28.56, 33.33, 40.44, 41.40, 45.67, 79.00, 114.25, 118.86, 119.83, 127.46, 138.18, 139.79, 151.04, 154.27, 154.35, 169.76; **LC-MS** (ESI) t_R = 9.22 min, 99 % purity, *m/z* $[M + H]^+$ calcd for $C_{26}H_{37}N_6O_3$, 481.3; found, 481.3.

***Tert*-Butyl (*S*)-3-(methoxy(methyl)carbamoyl)pyrrolidine-1-carboxylate (6)**

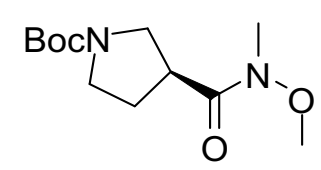

(*S*)-1-(*tert*-Butoxycarbonyl)pyrrolidine-3-carboxylic acid (**5**, 5.00 g, 23.2 mmol), *N*,*O*-dimethylhydroxylamine hydrochloride (2.53 g, 25.3 mmol), DMAP (0.14 g, 1.15 mmol), EDC × HCl (8.81 g, 46.0 mmol), and DIPEA (8.01 mL, 46.0 mmol) were dissolved in dry CH_2Cl_2 (30 mL) before stirring at rt for 18 h. Subsequently, EtOAc (200 mL) was added, and the organic layer was washed with saturated ammonium chloride solution (2 × 200 mL) and brine (200 mL), then dried over Na_2SO_4. Evaporation of the solvent *in vacuo* yielded an off-white oil that was used in the next step without further purification.

***Tert*-Butyl (*S*)-3-formylpyrrolidine-1-carboxylate (7)**

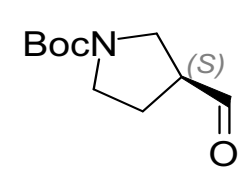

The crude product **6** was dissolved in dry THF (15 mL), and $LiAlH_4$ solution (1M in THF, 43.4 mL) was slowly added at 0 °C. After stirring for 1 h, the reaction was carefully quenched with *i*PrOH (50 mL), diluted with EtOH (300 mL), washed with brine (3 × 200 mL), dried over Na_2SO_4, and filtered through Celite. The resulting off-white, viscous oily residue was used without further purification.

***Tert*-Butyl (*R*)-3-((4-(4-((3-carbamoyl-6-(piperidin-1-yl)pyrazin-2-yl)amino)phenyl)piperidin-1-yl)methyl)pyrrolidine-1-carboxylate (8)**

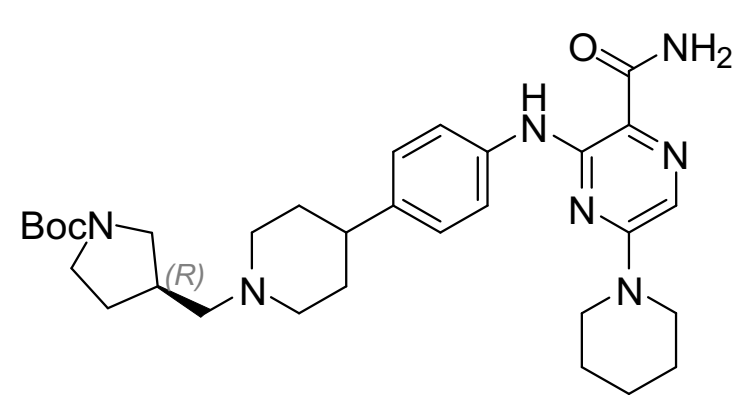

Compound **4** (7.69 g, 16.0 mmol) was treated with a solution of dry CH_2Cl_2 (10 mL) and trifluoroacetic acid (10 mL) at rt for 2 h. After evaporation of the volatiles and coevaporation with CH_2Cl_2 (3 × 10 mL), aldehyde **7** in dry THF was added (12.0 mmol), followed by the addition of sodium triacetoxyborohydride (6.80 g, 24.0 mmol) and sodium acetate (1.31 g, 16.0 mmol). Subsequently, the mixture was stirred at rt for 4 h. The final suspension was diluted with CH_2Cl_2 (300 mL), washed with brine (3 × 300 mL), and dried over Na_2SO_4. After filtration and evaporation of the volatiles, purification by flash chromatography on spherical silica gel (220 g, 30 µm, gradient from 0 to 5 % MeOH in CH_2Cl_2) afforded the title compound as a yellow solid.

Yield (2.72 g, 40 %); R_f = 0.39 (5 % MeOH in CH_2Cl_2); mp 200 – 205 °C (dec); **^{1}H NMR** (600 MHz, DMSO-d_6) δ 1.38 (d, *J* = 5.9 Hz, 9H), 1.54 – 1.75 (m, 10H), 1.94 (ddt, *J* = 10.1, 23.0, 45.4 Hz, 4H), 2.26 (d, *J* = 7.5 Hz, 2H), 2.33 – 2.46 (m, 2H), 2.90 (dd, *J* = 6.5, 14.8 Hz, 2H), 2.97 (s, 1H), 3.17 (t, *J* = 8.7 Hz, 1H), 3.30 – 3.39 (m, 2H), 3.66 (t, *J* = 5.4 Hz, 4H), 7.17 (d, *J* = 8.2 Hz, 2H), 7.28 (d, *J* = 2.9 Hz, 1H), 7.50 (d, *J* = 8.3 Hz, 2H), 7.64 (s, 1H), 7.70 (d, *J* = 2.9 Hz, 1H), 11.26 (s, 1H); **^{13}C NMR** (151 MHz, DMSO-d_6) δ 25.58, 28.65, 45.67, 54.29, 114.31, 118.79, 119.80, 127.42, 138.05, 140.30, 151.06, 154.01, 154.28, 169.76; **LC-MS** (ESI) t_R = 8.75 min, 99 % purity, *m/z* $[M + H]^+$ calcd for $C_{31}H_{46}N_7O_3$, 564.4; found, 564.6.

3-((4-(1-(((3*R*)-1-(2-(2,6-Dioxopiperidin-3-yl)-1,3-dioxoisoindolin-5-yl)pyrrolidin-3-yl)methyl)piperidin-4-yl)phenyl)amino)-5-(piperidin-1-yl)pyrazine-2-carboxamide (9, NX-2127)

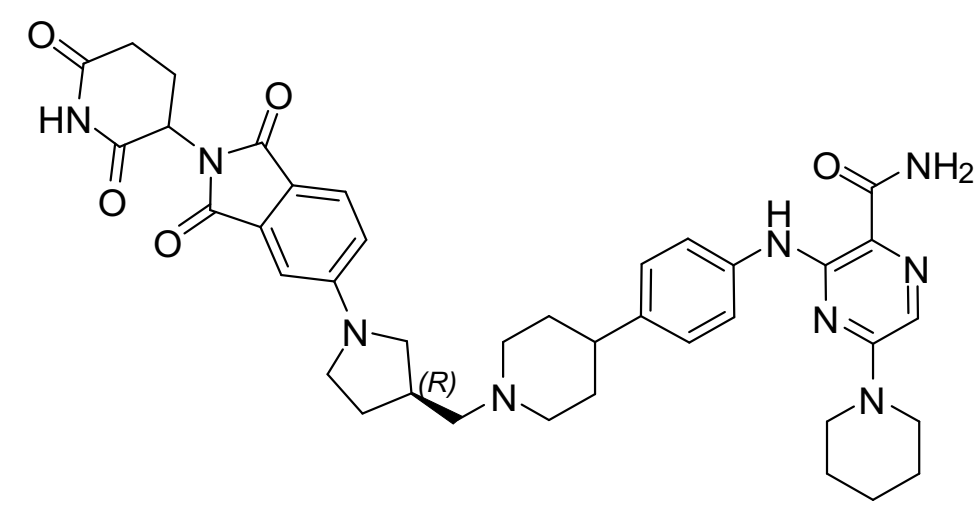

Compound **8** (2.72 g, 4.83 mmol) was treated with a solution of dry CH_2Cl_2 (10 mL) and trifluoroacetic acid (10 mL) at rt for 2 h. After evaporation of the solvents and coevaporation with CH_2Cl_2 (3 × 10 mL), the crude deprotected amine was dissolved in dry DMSO (20 mL) before 2-(2,6-dioxopiperidin-3-yl)-5-fluoroisoindoline-1,3-dione (1.60 g, 5.80 mmol) and DIPEA (2.52 mL, 14.5 mmol) were added. After stirring at 90 °C for 18 h and cooling to room temperature, CH_2Cl_2 (300 mL) was added, and the resulting solution was washed with brine (3 × 300 mL). The organic layer was dried over Na_2SO_4 and filtered. Purification by multiple flash chromatography runs on spherical silica gel (80 g, 15 µm, gradient from 0 to 6 % MeOH in CH_2Cl_2) yielded the title compound as an orange solid. The final product was extensively dried under high vacuum and elevated temperatures (50 °C) to remove solvent traces before further use for formulation studies.

Yield (1.08 g, 31 %); R_f = 0.47 (5 % MeOH in CH_2Cl_2); **^{1}H NMR** (600 MHz, DMSO-d_6) δ 1.55 – 1.70 (m, 6H), 1.80 – 2.05 (m, 4H), 2.17 (m, 2H), 2.28 (s, 1H), 2.50 – 2.61 (m, 3H), 2.76 – 2.79 (m, 1H), 2.87 (m, 2H), 3.04 – 3.07 (m, 2H), 3.36 – 3.43 (m, 1H), 3.49 – 3.72 (m, 8H), 3.78 (s, 1H), 5.05 (dd, *J* = 5.5, 12.7 Hz, 1H), 6.82 (d, *J* = 8.6 Hz, 1H), 6.93 (s, 1H), 7.19 (d, *J* = 8.1 Hz, 2H), 7.30 (s, 1H), 7.56 (d, *J* = 8.0 Hz, 2H), 7.64 – 7.68 (m, 2H), 7.72 (s, 1H), 10.68 (s, 1H), 11.04 (s, 1H), 11.31 (s, 1H); **^{13}C NMR** (151 MHz, DMSO-d_6) δ 22.69, 24.49, 25.58, 30.12, 31.43, 32.83, 45.68, 47.50, 49.16, 52.70, 52.84, 53.07, 55.34, 59.10, 106.03, 114.34, 115.80, 116.31, 118.98, 119.93, 125.43, 127.38, 134.45, 138.57, 151.01, 152.12, 154.26, 167.66, 168.12, 169.75, 170.56, 173.23; **LC-MS** (ESI) t_R = 7.96 min, 99 % purity, *m/z* $[M + H]^+$ calcd for $C_{39}H_{46}N_9O_5$, 720.4; found, 720.4.
